# Assessing the risk of resistance to cationic biocides incorporating realism-based and biophysical approaches

**DOI:** 10.1093/jimb/kuab074

**Published:** 2021-10-30

**Authors:** Laura J Fox, Paul P Kelly, Gavin J Humphreys, Thomas A Waigh, Jian R Lu, Andrew J McBain

**Affiliations:** Biological Physics, Department of Physics and Astronomy, Schuster Building, Faculty of Science and Engineering, University of Manchester, M13 9PL, UK; Division of Pharmacy and Optometry, School of Health Sciences, Faculty of Biology, Medicine and Health, The University of Manchester, Oxford Road, Manchester M13 9PT, UK; Division of Pharmacy and Optometry, School of Health Sciences, Faculty of Biology, Medicine and Health, The University of Manchester, Oxford Road, Manchester M13 9PT, UK; Biological Physics, Department of Physics and Astronomy, Schuster Building, Faculty of Science and Engineering, University of Manchester, M13 9PL, UK; Biological Physics, Department of Physics and Astronomy, Schuster Building, Faculty of Science and Engineering, University of Manchester, M13 9PL, UK; Division of Pharmacy and Optometry, School of Health Sciences, Faculty of Biology, Medicine and Health, The University of Manchester, Oxford Road, Manchester M13 9PT, UK

**Keywords:** Biocide, Resistance, AMR, Realism-based, Cross-resistance, Antibiotic resistance, Biophysics, Risk assessment, Membrane-targeted

## Abstract

The control of microorganisms is a key objective in disease prevention and in medical, industrial, domestic, and food-production environments. Whilst the effectiveness of biocides in these contexts is well-evidenced, debate continues about the resistance risks associated with their use. This has driven an increased regulatory burden, which in turn could result in a reduction of both the deployment of current biocides and the development of new compounds and formulas. Efforts to balance risk and benefit are therefore of critical importance and should be underpinned by realistic methods and a multi-disciplinary approach, and through objective and critical analyses of the literature. The current literature on this topic can be difficult to navigate. Much of the evidence for potential issues of resistance generation by biocides is based on either correlation analysis of isolated bacteria, where reports of treatment failure are generally uncommon, or laboratory studies that do not necessarily represent real biocide applications. This is complicated by inconsistencies in the definition of the term resistance. Similar uncertainties also apply to cross-resistance between biocides and antibiotics. Risk assessment studies that can better inform practice are required. The resulting knowledge can be utilised by multiple stakeholders including those tasked with new product development, regulatory authorities, clinical practitioners, and the public. This review considers current evidence for resistance and cross-resistance and outlines efforts to increase realism in risk assessment. This is done in the background of the discussion of the mode of application of biocides and the demonstrable benefits as well as the potential risks.

## Introduction

### Motivation, Scope, and Format

The current review seeks to address important points in microbial resistance to biocides that have been under-represented to date. The field is dominated with reports of “biocide resistance,” the definition of which is controversial, and correlation-based studies seeking to link biocide “resistance” to antibiotic resistance, often based on microbial strains that are forced to adapt to biocides in the laboratory. This review examines the use of the term resistance in the context of biocides and the relevance of laboratory derived microbial strains to real-world biocide application scenarios. It advocates for increased realism in assessing the risks from biocide use, to address the shortcomings of laboratory evolution studies. The focus is on cationic biocides as the class of primary concern in microbial resistance. The use of biophysical methods is discussed as an important tool for understanding the fundamentals of membrane–biocide interactions, important to promoting greater understanding and increased inter-disciplinary work in the field. A narrative style is adopted, focusing on representative studies to highlight the discussion points, and directing the reader to more in-depth studies and reviews as appropriate. Whilst the current review focuses on bacteria, it should be noted that concerns over antimicrobial resistance (AMR) also apply to fungi and viruses, as covered in a recent review by Meade and colleagues (Meade et al., 2021).

### Biocides in Context

The control of microorganisms is an important objective in many fields, including public health. The risks to health posed by the increasing problems of antibiotic resistance are well described and are a major threat to global health (Nathan, 2020; World Health Organisation, 2020). There is general agreement that the main driver of AMR is the use and misuse of antibiotics principally in human (Byrne et al., 2019; Palumbi, 2001) and veterinary practice (Patel et al., 2020), and there have been many initiatives to educate the public and practitioners about appropriate antibiotic use to preserve the limited antibiotic resource. Antibiotic resistance is an area of considerable global research activity both in terms of understanding the resistance mechanisms and towards developing strategies to combat the problem. Antibiotics are one of the few classes of drugs that commonly achieve a cure, but this effectiveness must be balanced against the problem of AMR (Nathan, 2020). The short duration of effective use and diminishing returns in new drug discovery have obstructed the new antibiotic development pipeline. Incentives for new antibiotic development and a better understanding of the factors that drive antibiotic resistance are therefore priority areas (Laxminarayan et al., 2016).

The implementation of practices that reduce infection and control microorganisms can directly reduce the need for antibiotic use and should be encouraged where possible. The use of non-antibiotic antimicrobial formulas, broadly termed biocides, is often an important route by which this can be achieved (Maillard, 2005). This is illustrated by the efficacy of hygiene, which revolutionised clinical practice in the 19th century and which remains relevant today (Gilbert & McBain, 2003; Maillard, 2018). However, restrictions on biocide production, often imposed through regulation, have limited the deployment of effective biocides and development of new biocidal products (Gilbert & McBain, 2003; Maillard, 2018). Against the background of rising antibiotic resistance and declining antibiotic development, restrictions on effective biocides may represent a risk to hygiene standards and our ability to control pathogenic microorganisms in the future.

### Biocides Defined

According to European legislation, biocides are defined as chemical substances or microorganisms (biological control agents) intended to destroy, deter, render harmless, or exert a controlling effect on any harmful organism (European Union, 1998). Most biocides are antimicrobial compounds and these fall into a range of chemical categories, that are deployed broadly in disinfectants, antiseptics and in some cases, preservatives. Whilst both antibiotics and biocides can be defined as antimicrobial compounds, the two groups of compounds are distinct for several important reasons. These include: (i) mode of action (MOA), where in contrast to the pharmacological specificity of antibiotics, biocides generally interact with multiple cellular targets, most notably cellular membranes (Gilbert & McBain, 2003; Maillard, 2018); (ii) concentration, where in contrast to the limited therapeutic window of antibiotics, when used as directed, biocides are generally present at concentrations considerably greater than the minimum bactericidal concentrations (Table 1) (Forbes et al., 2019; McBain et al., 2002); (iii) mode of delivery, where biocides are generally used in complex formulations (Forbes et al., 2017); and (iv) application, where biocides are not used systemically in humans or animals. The use of biocidal compounds for microbial control predates antibiotic use by many hundreds of years (Maillard, 2005). Biocides have applications that rival antibiotics in terms of importance through infection prevention, but their deployment extends into considerably more fields, including clinical antisepsis and disinfection, preservation in personal care products and industrial antifouling and clean-in-place.

**Table 1.** Cationic Biocide Concentrations Used in Various Applications, and Biocide Susceptibilities, Where Resistance or Reduced Susceptibility Has Been Reported

		In-use concentrations		
Application	Biocide	Figures reported	As μg ml^−1^	Examples of organism susceptibility
Hand scrubs; skin, mucosa, wound disinfection (China) (Liu et al., 2009; Kampf, 2018)	BAC	100–1000 mg l^−1^	100–1000	*Staphylococcus aureus*: 4 of 56 isolates (7%) with MBCs of 128 μg ml^−1^, exceeding only the minimum in-use concentration and contact time for mucosa/wound disinfection (100–500 μg ml^−1^, 5–180 min).
Surface disinfection (China) (Liu et al., 2009; Kampf, 2018)	BAC	1000–2000 mg l^−1^	1000–2000	52 of 56 isolates (93%) had MBCs ≤ 64 μg ml^−1^ for all conditions.
Surgical site, mucosa, wound antiseptic (Japan) (Narui et al., 2007; Kampf, 2018)	BAC chlorhexidine	100–500 mg l^−1^ 500–5000 mg l^−1^	100–500 500–5000	*S. aureus* vs. BAC: a few (of 42) isolates had MBCs over 100 μg ml^−1^ at 5 min exposure, exceeding minimum in-use concentration and contact time for surgical site/mucosa/wound disinfection. Vast majority of isolates had MBCs ≤ 64 μg ml^−1^ for all conditions.
Hand scrub, instrument disinfection (Japan) (Narui et al., 2007; Kampf, 2018)	BAC chlorhexidine	500–1000 mg l^−1^ 1000–5000 mg l^−1^	500–1000 1000–5000	*S. aureus* vs. chlorhexidine: a few (of 42) isolates had MBCs over 500 μg ml^−1^, exceeding minimum in-use concentration and contact time (500 μg ml^−1^, 5–180 min) for mucosa/wound and surface disinfection. Vast majority of isolates had MBCs ≤ 256 μg ml^−1^ for all conditions.
Surface disinfection (Japan) (Narui et al., 2007; Kampf, 2018)	BAC chlorhexidine	500–2000 mg l^−1^ 500 mg l^−1^	500–2000 500	
Veterinary environmental treatment (Europe) (Couto et al., 2013; Kampf, 2018)	BAC	60–120 mg l^−1^	60–120	*Staphylococcus* spp. from horses: 5 of 14 isolates with BAC MBCs > 128 μg ml^−1^ were found. Overall, MBC values were lower than concentrations used in commercial products.
Veterinary surgical site treatment (Europe) (Couto et al., 2013; Kampf, 2018)	BAC	100–500 mg l^−1^	100–500	3 *Staphylococcus* spp. from infected horses (Bjorland et al., 2003): highest MIC for BAC was 6 μg ml^−1^.
Veterinary hand scrub (Europe) (Couto et al., 2013; Kampf, 2018)	BAC	500–1000 mg l^−1^	500–1000	
Veterinary skin and wound treatment (Europe) (Couto et al., 2013; Kampf, 2018)	BAC	1000–2000 mg l^−1^	1000–2000	
Swimming pools and water displays (USA) (United States Environmental Protection Agency, 2004b, 2006b, 2006a)	BAC DDAC PHMB	1.2–6.2 ppm 0.5–2.0 ppm 30–50 ppm	1.2–6.2 0.5–2.0 30–50	No reports found.
Egg shell sanitising (USA) (United States Environmental Protection Agency, 2006b, 2006a)	BAC	200 ppm	200	*E. coli* (27 isolates) and *Salmonella* spp. (39 isolates) (Spain): hen egg shell isolates from 2 studies (Grande Burgos et al., 2016; Fernández Márquez et al., 2017) reported maximum MICs of 75 μg ml^−1^ (*E. coli*) and 100 μg ml^−1^ (*Salmonella*) for BAC. MBCs not reported.
Agricultural premises and equipment (by mop, spray, swab, immersion, USA) (United States Environmental Protection Agency, 2004a, 2004b, 2006b, 2006a)	BAC DDAC PHMB	2036 ppm 786–1120 ppm 0.56%	2036 786–1120 5600	*S. aureus (MRSA)*: 40 isolates from nursery pigs (Ontario, Canada) (Slifierz, Friendship and Weese, 2015). Maximum MICs reported for BAC were 5 μg ml^−1^.
Medical premises and equipment (by mop, spray, swab, immersion, USA) (United States Environmental Protection Agency, 1984, 2004b, 2006b, 2006a)	BAC DDAC PHMB	2080 ppm 786–2383 ppm 0.56–3.17%	2080 786–2383 5600–31 700	*S. aureus*: 94 clinical isolates (UK) (Smith and Hunter, 2008). Highest MBCs were < 100 μg ml^−1^ for a commercial quaternary ammonium biocide (Trigene) recommended for surface disinfection at 1000 μg ml^−1^. *Pseudomonas aeruginosa*: 124 clinical isolates (Brazil) (Romão et al., ww^2011^). 46% of isolates had reduced susceptibility versus reference strain (AOAC use-dilution method). A hospital disinfectant with 2000 μg ml^−1^ BAC was used. *Salmonella* Enteritidis and *S.* Typhimurium clinical isolates (UK) (Braoudaki and Hilton, 2005): Parent strains MIC for BAC was 32 μg ml^−1^, up to 256 μg ml^−1^ for lab-adapted strains.
Food handling/storage premises and equipment disinfection (USA) (United States Environmental Protection Agency, 1984, 2004a, 2004b, 2006b, 2006a)	BAC DDAC PHMB	2036–2080 ppm 2383 ppm 100 ppm–3.17%	2036–2080 2383 100–31 700	*Salmonella* isolates including food isolates (Condell et al., 2012): Maximum MICs were between 3.13% and 25% of use concentrations for commercial food industry QAC and/or biguanide formulations. Maximum MIC for BAC, lab-adapted strain was 94 μg ml^−1^. Maximum MIC for chlorhexidine, lab-adapted strain > 1000 μg ml^−1^. 378 isolates, various species, from organic foods (Fernández-Fuentes et al., 2012): Maximum MICs of 100 μg ml^−1^ (BAC and DDAC), 1000 μg ml^−1^ (cetrimide, hexadecylpyridinium chloride), 5000 μg ml^−1^ (chlorhexidine). *Listeria monocytogenes*, 392 isolates from food and clinical sources (Meier et al., 2017): Maximum MICs for BAC > 30 μg ml^−1^. 1237 bacterial isolates, various species, from a slaughterhouse (Spain) (Lavilla Lerma et al., 2013): isolates were defined as “resistant” by growth in the presence of 0.025 μg ml^−1^ of various biocides, including hexadecylpyridinium chloride, cetrimide, PHMG, BAC, and chlorhexidine.
Public eating places, dairy processing equipment (USA) (United States Environmental Protection Agency, 2006b, 2006a)	BAC DDAC	200 ppm 200 ppm	200 200	
Food-processing equipment and utensils (USA) (United States Environmental Protection Agency, 2006b, 2006a)	BAC DDAC	200–400 ppm 200–400 ppm	200–400 200–400	
Commercial, institutional, and industrial premises and equipment (by mop, spray, swab, immersion, USA) (United States Environmental Protection Agency, 2004a, 2004b, 2006b, 2006a)	BAC DDAC PHMB	596–2980 ppm 786–2383 ppm 0.56%	596–2980 786–2383 5600	*Acinetobacter, Citrobacter*, and *Pseudomonas* industrial isolates (Lear et al., 2006): MICs up to 340 μg ml^−1^ for BAC and 85 μg ml^−1^ for chlorhexidine.
Residential and public access premises (by mop, spray, swab, immersion, USA) (United States Environmental Protection Agency, 1984, 2004a, 2004b, 2006b, 2006a, 2009)	BAC DDAC PHMB	596–2980 ppm 512–1311 ppm 0.1–3.17%	596–2980 512–1311 1000–31 700	*Staphylococcus* spp. (653 isolates) from surfaces of weight machines, exercise bikes, dumbbells, boxercise gloves, refrigerator door handles, toilet handles, television remote controls and bathroom sink handles (He et al., 2014): 63 isolates had MICs > 3 μg ml^−1^ and therefore reported as BAC resistant. Domestic drain (18 isolates) bacteria: MBCs after lab adaptation to biocides (Moore et al., 2008): 3.9–187 μg ml^−1^ (QAC1, DDAC), 31.2–292 μg ml^−1^ (QAC2, BACs mixture), 7.8–250 μg ml^−1^ (chlorhexidine), 3.9–208 μg ml^−1^ (PHMB). Culture collection, human skin, and domestic drain bacteria (18 species): MBCs after lab adaptation/reversion in presence/absence of biocides (Forbes et al., 2014): 7.3–464 μg ml^−1^ (cetrimide), 7.3–116 μg ml^−1^ (chlorhexidine), 7.3–116 μg ml^−1^ (PHMB).
Carpets (hospitals, homes, commercial, institutional, and industrial premises, USA) (United States Environmental Protection Agency, 2006b, 2006a)	BAC DDAC	16 800 ppm 12 150 ppm	16 800 12 150	
Wound antisepsis and decontamination (Eberlein and Assadian, 2010; Kampf, 2018; Machuca et al., 2019)	PHMB chlorhexidine	0.02–0.5% 2%	200–5000 20 000	Multidrug resistant, biofilm-forming bacteria (8 strains) (Machuca et al., 2019): MBCs for PHMB-B (0.1% PHMB, 0.1% betaine): 1–8 μg ml^−1^. MBCs for chlorhexidine (2%): 4–32 μg ml^−1^.
Hand scrub (Kampf, 2016, 2018)	chlorhexidine	0.1–4%	1000–40 000	*S. aureus* (38 hospital acquired, 25 community MRSA) (Smith and Hunter, 2008): Mean MBCs: < 0.01% (Trigene, mixture of BAC, DDAC, use conc. 0.1%), < 0.004% (MediHex-4, chlorhexidine, use conc. 4%). *S. aureus* (MRSA) and * aeruginosa* planktonic and biofilm cultures (Smith and Hunter, 2008): MBC ranges for *S. aureus*: 0.0004–0.001% (Anticide Bac-50, BAC, use conc. 1%, and MediHex-4, chlorhexidine, use conc. 4%). MBC ranges for *P. aeruginosa*: 0.01–0.1% (Anticide Bac-50) and 0.4–2% (MediHex-4). Biofilm survival was high with in-use concentrations. High chlorhexidine resistance rates (MICs) reported (Kampf, 2016) in *Enterobacter* spp. (10–75 μg ml^−1^), *Pseudomonas* spp. (2–800 μg ml^−1^), *Proteus* spp. (10–1600 μg ml^−1^), *Providencia* spp. (500–1600 μg ml^−1^) and *Enterococcus* spp. (2–2500 μg ml^−1^), and strong ability of *Acinetobacter* spp., *Klebsiella pneumoniae* and *Pseudomonas* spp. to adapt to chlorhexidine.
Surgical site antiseptic (Kampf, 2016, 2018)	chlorhexidine	0.1–2%	1000–20 000	
Mucosa and wound antiseptic (Kampf, 2016, 2018)	chlorhexidine	0.05%	500	
Surface disinfectant (Kampf, 2016, 2018)	chlorhexidine	0.05%	500	
Instrument disinfectant (Kampf, 2016, 2018)	chlorhexidine	0.1–0.5%	1000–5000	
Antibacterial mouthwash (Cieplik et al., 2019)	chlorhexidine	0.2%	2000	

BAC, benzalkonium chloride; DDAC, didecyldimethylammonium chloride; chlorhexidine, Chlorhexidine; PHMB, polyhexamethylene biguanide.

Biocides fall into different chemical classes, including alcohols, chlorine or chlorine-releasing compounds, peroxides, aldehydes, and phenolic compounds. A more in-depth discussion of these classes and their modes of action can be found elsewhere (Jones & Joshi, 2021; Kampf, 2018). The current review focuses on the cationic biocides, including Quaternary ammonium compounds (QACs) and biguanides, as these are amongst the most widely used biocidal compounds, and have become a focus of concern for potential resistance generation. These compounds differ fundamentally from the majority of antibiotics in their MOA and interaction with the microbial cell, generally having multiple concentration-dependent targets most notably cell membranes, as opposed to the pharmacological specificity of antibiotics (Gilbert & McBain, 2001). As consumer awareness of hygiene increases, so does the global market for biocides in antiseptic and disinfectant products, predicted to reach $8.1 billion by 2021 (Zion Market Research, 2016). The wide range of medical, industrial and domestic uses for biocides is illustrated below.

### Common Applications of Biocides

Biocides, including bisbiguanides QACs, have broad applications in healthcare, agriculture, food industry, cosmetics and domestic cleaning. In healthcare applications, biocides are used either as disinfectants in decontamination of medical devices, surfaces and intact skin, or as antiseptics applied to skin and mucosa (SCENIHR, 2009). Sterilisation of critical devices, such as surgical instruments, needles and syringes, implants and devices inserted into the vascular system or urinary tract, is often achieved during manufacture, by steam under pressure. However, chemical disinfection may be used where the device is heat sensitive. Similarly, for semi-critical devices such as probes and endoscopes, high-level disinfection is commonly employed (SCENIHR, 2009). Devices that contact intact skin, such as stethoscopes, bedpans and blood pressure cuffs, have a low risk of infection and are considered suitable for disinfection. In the United States the FDA have approved quaternary ammonium detergent solutions as clinical disinfectants, with high-level disinfection limited to aldehydes, hydrogen peroxide, peracetic acid, and hypochlorite (SCENIHR, 2009). Disinfection of wounds or intact skin and mucosa is often carried out to reduce or eliminate pathogenic bacteria (or bioburden). Biocides including various QACs and chlorhexidine gluconate are also used (SCENIHR, 2009). Chlorhexidine is the most widely used biguanide biocide, deployed in formulations of hand-washes, surgical scrubs, bathing decolonisers, and wound dressings (McDonnell, 2017). The biguanides chlorhexidine and octenidine are also used to decolonise patients known to carry methicillin-resistant *Staphylococcus aureus* (MRSA) (Hardy et al., 2018), and the polymeric biguanide polyhexanide (PHMB) is an effective wound antiseptic (Hübner & Kramer, 2010). Within the farming and food-processing industries, biocides are used extensively in preservation of fresh foods, and in disinfection of surfaces, holding pens, livestock, and processing equipment. As AMR is a recognised problem in farming and food production, there has been much focus on the potential role of biocide use in promoting cross-resistance to antibiotics, as reviewed in detail elsewhere (Davies & Wales, 2019; Giacometti et al., 2021). QACs and cationic biocides have featured prominently in such research due to their widespread application in this sector (see Table 1). Biocides for domestic cleaning are utilised in cleaning products, laundry detergents, and general disinfectants. QACs do not generally require post-application rinsing and as such are formulated into sprays, wipes, and concentrates for use on horizontal surfaces, walls, and floors (McDonnell, 2017). In a Danish Environmental Protection Agency report on industrial and institutional cleaning products, a limited number of biocides were identified, accounting for around 15% of the 275 ingredients described (cationic surfactants accounted for 9%), with 65% being surfactants (Madsen T, 2005; SCENIHR, 2009). Domestic cleaning products might be expected to have similar ingredient profiles. Biocides are commonly used as preservatives in personal care and consumer products (Gilbert & McBain, 2003; Maillard et al., 2013). In the EU, Article 2 of Regulation 1223/2009 on cosmetic products (The European Parliament and the Council of the European Union Regulation no 1223/2009 on cosmetic products, 2009) defines a cosmetic product as “any substance or mixture intended to be placed in contact with the external parts of the human body (epidermis, hair system, nails, lips and external genital organs) or with the teeth and the mucous membranes of the oral cavity with a view exclusively or mainly to cleaning them, perfuming them, changing their appearance, protecting them, keeping them in good condition or correcting body odours,” which encompasses a wide range of products including shampoos, conditioners, shower gels, creams and lotions, toothpaste, mouthwashes, and deodorants to name but a few. Of the 57 chemicals listed in Annex V of the directive as permitted in cosmetic products (within the limits defined therein), several are cationic biocides including chlorhexidine, polyaminopropyl biguanide, cetrimonium bromide/chloride, benzalkonium chloride (BAC), and benzethonium chloride (European Union, 2009; SCENIHR, 2009).

The above illustrates how biocides have found applications in many important areas of human activity. In this context, their effectiveness in the control of microbial growth, and the wider implications of their use, should be a matter of ongoing investigation.

## Measuring Microbial Susceptibility to Biocides

There are several ways of assessing the susceptibility of a bacterium to a biocidal compound, which can be broadly applied to both cationic and other molecules. The extent to which a test represents the real use of a biocide often increases with the complexity of the method. Many biocide studies, including where resistance is reported, have used minimum inhibitory concentrations (MICs) and minimal bactericidal (or biocidal) concentrations (MBCs) as the main metrics for comparison due to their ease of use and reproducibility (McDonnell & Russell, 1999). The analogous minimal fungicidal concentration (MFC) test can be applied when studying fungi (Espinel-Ingroff et al., 2002). The MIC is probably the most used test in biocide studies (Ahn et al., 2016; Aiello et al., 2004) but is arguably one of the least appropriate. With some exceptions, biocides are applied to inactivate or kill microorganisms, not to prevent growth. The MBC test can be modified by including substrata materials upon which biofilm can be grown, to determine the minimum biofilm eradication concentration (MBEC) (Stoodley, 2016). These methods respectively define an endpoint based on the minimum concentration of an antimicrobial compound that either prevents microbial growth or sterilises planktonic, or sessile microbial cultures. In most cases for these methods, the antimicrobials are applied in doubling dilutions such that where a change in susceptibility is reported the minimum difference that could be detected is a factor of two. Other methods include disk or well diffusion, where a biocide generates a concentration gradient across solid bacteriological media, inhibiting the growth until the concentration becomes low enough to permit growth. Arguably the most appropriate approach for biocide susceptibility testing involves challenge tests that determine inactivation dynamics by applying the biocide to a culture of known microbial density and measuring decreases in bacterial viability over time. This can also be expressed as the lowest concentration achieving a set number of decimal reductions over time, using standardised methods. Challenge tests can also be extended to test biofilms using multiple plate wells in batch or fed batch systems or continuous culture, for example, using the constant depth film fermenter (CDFF) (McBain, 2009). In all the above methods there may be factors present in real application of biocides that are not reproduced by the test method including the growth of microorganisms in complex communities (Forbes et al., 2017), growth (Evans et al., 1991), formulation (Forbes et al., 2016, 2017), duration of exposure (McDonnell & Russell, 1999), and presence of organic soil.

## Modes of Action of Cationic Biocides

For most antimicrobials, the first step in their action is an interaction with or penetration through the cell membrane. A target of some antimicrobials is the further disruption of this membrane either by physical interactions affecting overall structure and stability or by specific interactions with lipid or protein targets or precursors to their synthesis. However, the action of antimicrobials that cause direct or indirect disruption of cellular membranes may extend beyond the membrane to interactions with cytoplasmic constituents. Here we will discuss the action of only one class of antimicrobials which are commercially available cationic biocides, however, antimicrobials in general can have specific and/or non-specific interactions with bacteria.

As discussed later in this article, much of the biocide resistance reported in the literature, especially for cationic biocides, is attributed to efflux activity. Whilst such mechanisms may protect cytoplasmic constituents, it is unclear how pumping biocides out of the cell might prevent disruption of the outer membrane. Therefore, understanding the interactions that take place between cationic biocides and cell membranes is of vital importance in understanding their MOA, mechanisms underlying susceptibility, and structure–function relationships for the improved intelligent design of these biocides in the future.

### Mode of Action of QACs

**Fig. 1.**
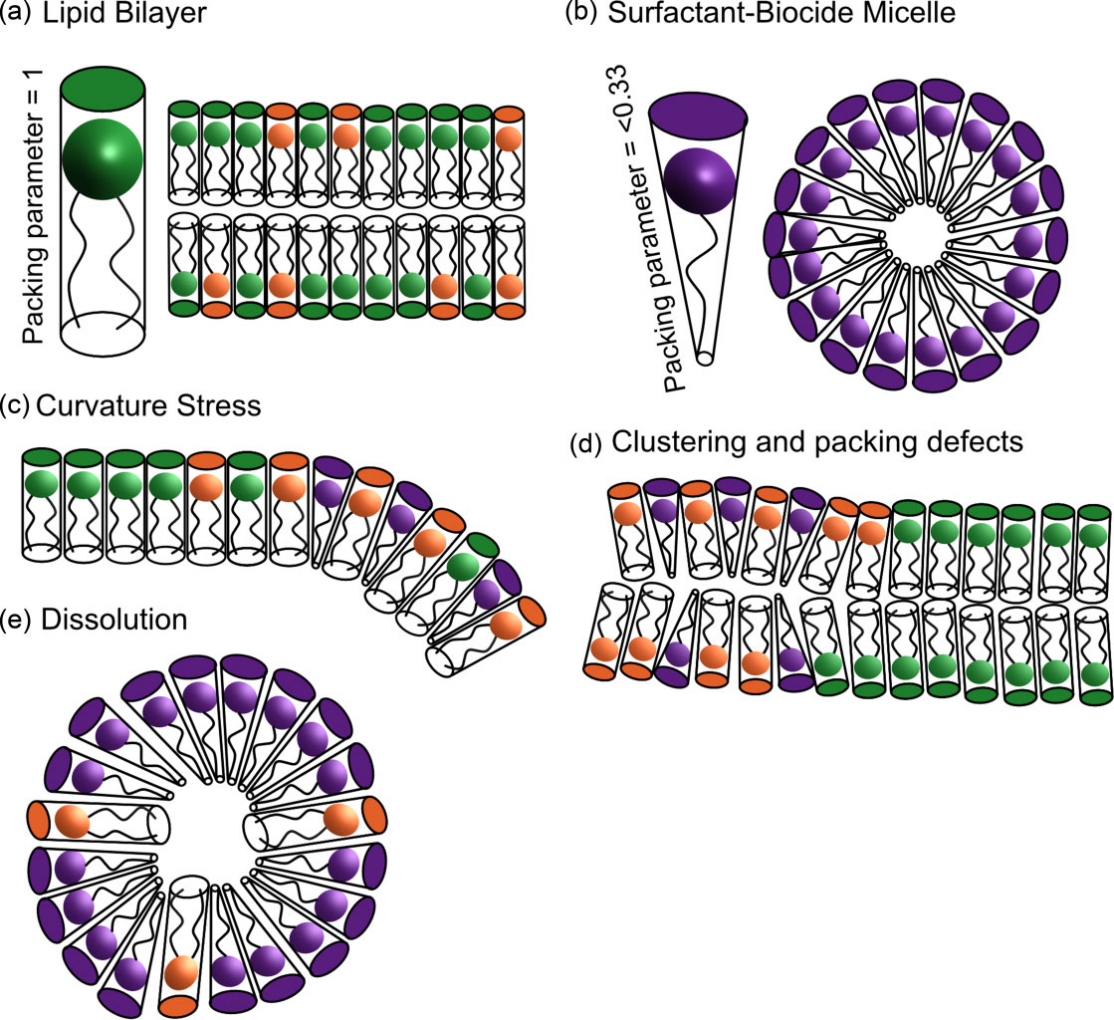
Surfactant membrane interactions. (a) Formation of lipid bilayer by phospholipids. (b) Formation of micelle by a surfactant-biocide. The interaction of surfactant-biocide molecules with lipid bilayers (orange; anionic lipids, green; zwitterionic lipids) disrupts ordering and structure via curvature stress (c), clustering of charged lipids and packing defects (d), and dissolution (e).

The antimicrobial action of QACs was characterised by the rapidity of action and cell lysis as early as the 1950s (Salton, 1951). QACs affect the bulk physical properties of cell membranes, such as membrane structure and integrity (Wessels & Ingmer, 2013) (see Fig. 1), unlike some antimicrobial peptides and antibiotics which have specific molecular targets (Epand et al., 2016). Even with the apparent non-specificity of QACs, there are some examples of bacteria exhibiting comparatively lower sensitivities to these compounds and the presence of QAC-resistance genes which will be considered later in this article.

Despite the long-term usage of QACs, the membrane disruptive mechanisms of these biocides have not been fully elucidated. Considerable differences have been observed in the MICs of cationic-surfactant biocides which is thought to depend upon their hydrophobic chain length/hydrophobicity, the number of cationic groups or the amount of chain branching. To further understand the difference in susceptibility associated with small changes in molecular structure, a structure–function relationship needs to be elucidated to further the understanding of the MOA of these compounds and progress towards this goal using biophysical approaches are discussed in further detail in subsequent sections.

### Mode of Action of Chlorhexidine

Parallels can be drawn between QACs and other cationic biocides, such as chlorhexidine. It has long been known that chlorhexidine causes deformation to bacteria membranes, however, there have previously been conflicting studies claiming the major MOA of chlorhexidine is the inhibition of ATPases. Localised morphological damage to *Bacillus subtilis* and *Escherichia coli* membranes alongside biochemical and protein analysis by Cheung et al. indicate that chlorhexidine acts on the differentially distributed lipids in bacteria cell membranes (Cheung et al., 2012).

A recent biophysical study attempting to further unravel the molecular mechanisms of action of chlorhexidine indicates the composition and therefore stiffness of cell membranes also plays a vital role in the selectivity of chlorhexidine rather than electrostatic interactions alone. MD simulations show changes in membrane packing in the presence of chlorhexidine, indicating more discrete changes in membrane properties which may lead to impaired function (Rzycki et al., 2021). These studies again reiterate the importance of understanding how the physical properties of biocides and cell membranes affect their interactions and the subsequent biocidal effects.

### The Mode of Action of Polymeric Guanidines

Polymeric guanidines such as polyhexamethylene guanidine (PHMG) and polyhexamethylene biguanide (PHMB), are biocidal polymers made with repeating guanidine or biguanidine units, connected by hydrocarbon chains that can be made with varying charge and alkyl chain length. They are used widely for the treatment of swimming pools and as antiseptics and usually are characterised by a high therapeutic index due to low activity against mammalian cells compared to bacteria.

The MOA of polymeric guanidines is thought to be strong binding to negatively charged phospholipid membranes (Ikeda, Tazuke, & Watanabe, 1983) followed by subsequent perturbations of the polar headgroups and hydrophobic core region of the phospholipids membrane (Zhou et al., 2011). Despite interesting biophysical studies by Zhou et al. linking hydrophobic chain length and membrane disruption, there remains a debate on the MOA of these polymers. Like the QAC-surfactants, the MOA of PHMB has long been considered to be dominated by membrane disruption, however, recent studies also report the ability of PHMB in particular, to bypass the cell membrane and to condense bacterial chromosomes (Chindera et al., 2016; Sowlati-Hashjin et al., 2020).

## Biophysical Approaches to Studying Membrane–Biocide Interactions

**Fig. 2.**
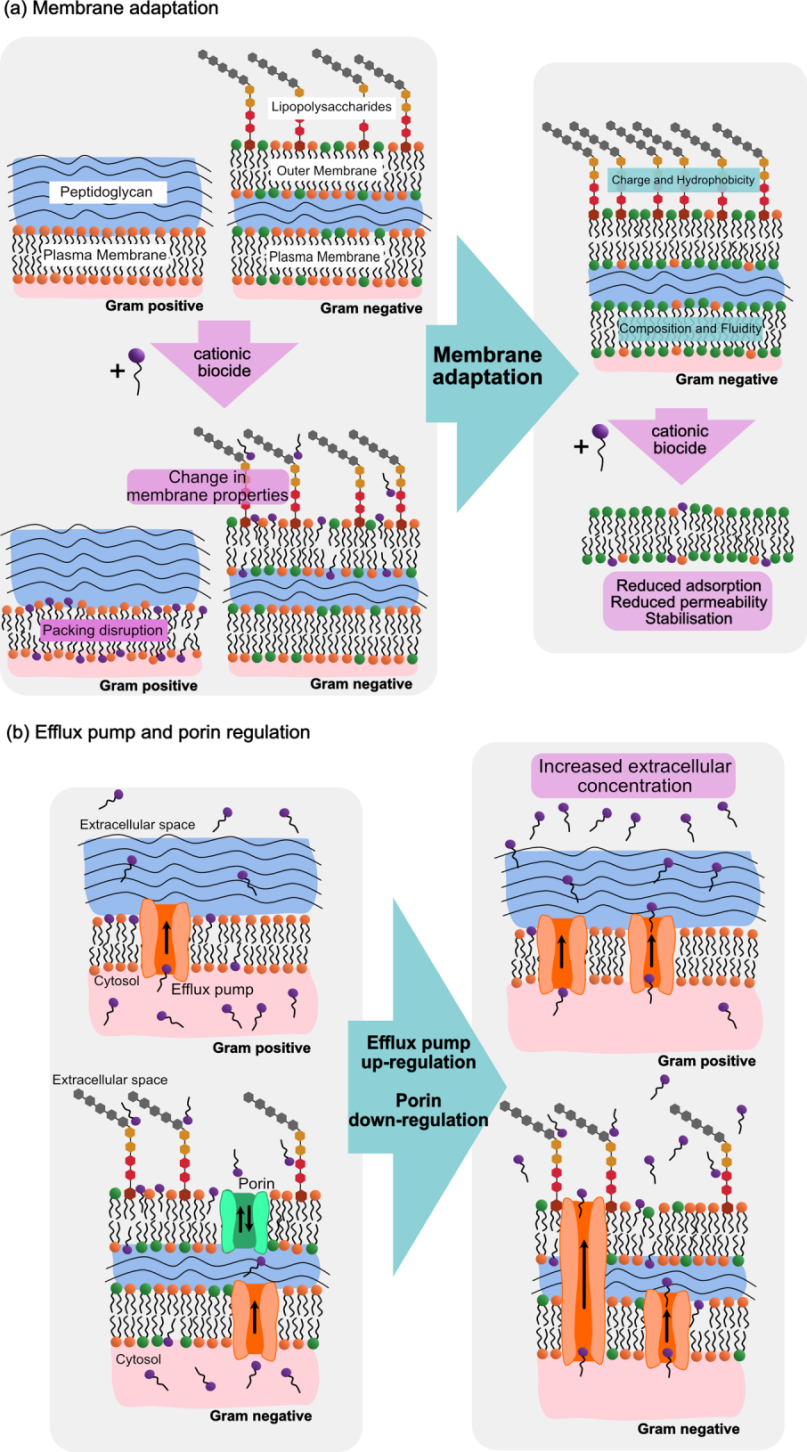
Adaptations to cationic biocides. (a) Membrane adaptations include changes in lipid composition and fluidity that reduce biocide adsorption and intercalation in both gram-positive (not shown) and gram -negative bacteria. These adaptations include changes in lipid charge and alkyl chain length (represented by increase in green-coloured lipids) or changes in LPS composition in outer membrane of gram-negative bacteria. (b) Some efflux pumps work consistently in cells, adaptation to biocides could include upregulation of efflux pumps expelling more biocide molecules from the cytoplasm and downregulation of porins.

Understanding the MOA of biocides in complex organisms can involve measurements of changes in membrane integrity by leakage of cellular constituents into extracellular environment, changes in cellular pH and changes in ATP synthesis. However, these measurements do not allow access to the exact molecular mechanisms occurring between antimicrobials and cellular membranes due to the nature of the complex biological environment. The use of model membranes is therefore preferable to study these molecular details and to understand how the physicochemical properties of a biocide affect interactions with bacterial membranes and allude to a structure–function relationship between biocidal properties, MOA, potency and hence resistance through membrane adaptation as discussed later in the review (see Fig. 2).

There are several model membranes that are commonly utilised and usually include the use of either a single leaflet of a membrane (monolayer) or both leaflets (bilayer) and can include singular or multiple components, such as different species of phospholipids. Membrane models include, but are not limited to: liposomes, supported lipid bilayers, lipid monolayers, and lipid mesophases (Pomorski et al., 2014; Yeagle, 2016). The physical properties of these models, such as their size, structure and fluidity, in the absence and presence of antimicrobial compounds can be studied with a range of surface/interface sensitive and bulk measurement techniques. Model membrane studies often appear to neglect large portions of the larger membrane structure of bacteria, most often to establish a balance between comparability to real biological systems and the required simplicity to obtain detailed structural and conformational information on individual components. Usually, bacterial membranes are modelled considering only the phospholipids as the vital structural components of both gram-negative and gram-positive bacteria (Alkhalifa et al., 2020). In reality, components such as integral proteins and peptidoglycan play a vital role in the structure, dynamics and function of the cell membrane as well as the wider environment (see Fig. 2). Therefore, it is important to consider the effects of these additional components on membrane properties and interactions with biocides when using more simplistic models.

## Factors Influencing the Activity of Cationic Biocides: Intrinsic Physicochemical Properties

Surfactant biocides, such as QACs, have defined physical properties such as critical micelle concentrations (CMCs), surface tension at CMC and solubility boundaries. QACs are largely non-specific and disrupt the overall physicochemical properties of the membrane such as structure (curvature, clustering, and defects), fluidity, permeability, and charge eventually resulting in cell lysis or the solubilisation of membrane lipids into micelles (Epand et al., 2016) (see Fig. 1). These effects are not independent, as changes in membrane curvature will also affect fluidity and cause packing defects (and *vice versa*), as described by Epand et al. in another review (Epand et al., 2016).

As suggested above, interactions between biocides and cell membranes can vary depending on the intrinsic physical properties of the biocide, such as chain length and branching, as well as extrinsic factors such as the composition of the bacterial membrane and the wider chemical environment. An important intrinsic feature of many membrane-targeted biocides is their amphipathic nature. Surfactant biocides, such as didecyldimethylammonium chloride (DDAC), have hydrophobic alkyl chains allowing for insertion into the hydrophobic core of cell membranes whilst quaternary ammonium cations interact with anionic lipid headgroups, particularly prevalent in bacterial membranes. These intrinsic properties of the biocides result in several variable physical characteristics such as the packing parameter (determined by the size and shape of the hydrophobic and hydrophilic portions of the molecule), CMC and surface tension at CMC.

### Packing Parameter

The overall shape or packing parameter (*P_c_*) of an amphipathic molecule is dependent upon the volume and area of hydrophobic and hydrophilic portions of the molecules and gives rise to an intrinsic curvature affecting the types of secondary structures it can form (Israelachvili, 2011). Surfactants, such as dodecyltrimethylammonium bromide (DTAB), often have cone-like shapes with packing parameters <0.33 and positive intrinsic curvature, this results in the formation of micelles in solution at a critical concentration of the surfactant (CMC) (Fig. 1b).

Phospholipids that make up the primary structure of cellular membranes have cylindrical-type shapes and packing parameters close to 1 (Fig. 1a). Phospholipids can therefore, form lamellar structures spontaneously, a property that allows for the ease of membrane formation for model systems. Some anionic phospholipids, such as phosphatidylethanolamines (PE) present in bacterial membranes, have slight negative curvature and an inverse cone shape due to their comparatively smaller headgroups. Whereas others such as cardiolipin (CL) have a wedge shape and large negative intrinsic curvature, due to the small head group cross-section relative to the cross-section of the four large tail groups. CL is localised at the poles of *E. coli*, with theories suggesting this is a curvature-mediated microphase separation (Oliver et al., 2014). Insertion of a molecule, such as DTAB, with mismatched positive intrinsic curvature into a lamellar cellular membrane, could cause changes in curvature, packing defects, alter the fluidity of the membrane and impair membrane function (Fig. 1c–e) (McMahon & Boucrot, 2015).

### Hydrophobicity, Chain Length, and Branching

The length of the hydrophobic chain of a surfactant molecule influences its solubility and the concentration at which it forms micelles in solution (CMC) as discussed above. The chain length and hydrophobicity can also be correlated with the potency of the biocide. For example, Inácio et al. compared the CMC of C_10_-C_16_ TAB QACs (cetyltrimethylammonium bromides [CTABs]) with their MIC, to relate molecular structure to biocidal potency (Inácio et al., 2016). Overall, a decrease in CMC and MIC was observed with increasing alkyl chain length (or increasing hydrophobicity). Further to this, C_10_TAB and C_12_TAB inhibited bacterial cell growth at concentrations much less than their CMC, whereas for the longer-chained C_14_TAB and C_16_TAB growth was only inhibited close to or above CMC. This large disparity in the MICs relative to the CMCs was suggested to be due to differences in the MOA. C_10_TAB and C_12_TAB were considered to exert their antimicrobial effects by altering membrane properties such as elasticity or porosity and C_14_TAB and C_16_TAB could cause cell leakage, gross membrane disarrangement or dissolution. Although MIC and MBC are a common measure of the antimicrobial activity of a substance it does not offer any mechanistic insight into its fundamental interactions with bacteria. Inácio and colleagues assessed biocide toxicity at different concentrations by exposure of bacterial cultures followed by serial dilution and colony counting. Three stages of toxicity to the studied QACs were thus observed (Inácio et al., 2016). The following three MOAs were identified at different concentrations: “impairment of energetics and cell division at low concentrations; membrane permeabilisation and electron transport inhibition at medium concentrations; and disruption of bacterial membranes and cell lysis at high concentrations” (close to the CMC).

Gemini-TAB surfactants interacting with DPPC: Chol (dipalmitoyl phosphatidylcholine-cholesterol) liposome membrane models were also found to cause membrane disruption dependent upon the length of the acyl chains of the TAB and the spacer length between the head-groups (Almeida et al., 2011). It was found that surfactants with chain lengths similar to the phospholipid chain lengths caused less disruption to the membrane structure than those with shorter chains, which may indicate why a larger fraction of the longer-chain surfactants is required to cause membrane disruption sufficient to inhibit cell growth.

Further morphological studies using ^31^P NMR and molecular dynamics (MD) simulations indicated that differences in the disruption caused by varying chain and spacer lengths could be caused by packing differences in the membrane (Almeida et al., 2011). The length of hydrophobic components influences their shape upon insertion into membranes and therefore the membrane curvature and packing of the lipid molecules (Fig. 1). Cationic-surfactant biocides with curvatures closer to that of the membrane lipids may result in less disruption. An alternative explanation could be that the low CMC of long-chained gemini surfactants causes the efficacy to be compromised, since it is likely that aggregates will be interacting with the membrane rather than individual molecules, impacting the biocides’ ability to insert into the membrane and cause disruption.

### Charge

The cationic moiety architecture, charge density, and counter anions can influence biocide interactions with cell membranes. It has been long known that the antimicrobial efficacies of cationic biocides are affected by the associated counter anions due to varying dissociation ability of the molecules and anion- dependent varied structure of the biocides. Counter ions with higher dissociability allow better contact with the cell membrane. A study on the effect of seven different counter anions on the activity of a cationic polymer (poly(4-vinyl pyridine) revealed changes in polymer morphology and –OH counterions resulting in the lowest MIC values against strains studied (Sharma et al., 2010).

The counterion also influences the hydrophobicity and therefore solubility of the biocides which effects their biological activity. This can also be linked to extrinsic effects such as the presence of additional ions in the environment, which cause changes in the solubility of biocides as well as effects such as screening charge interactions with negatively charged bacteria membranes (Crismaru et al., 2011).

There is also evidence to suggest that there is a charge-density threshold, above which bacteria death occurs quickly (Kügler et al., 2005). Although these studies are undertaken on substrates bearing quaternary ammonium groups, the same principles could apply generally for cationic biocides, with the killing effect being related the ion exchange between the bacterial membrane and quaternary ammonium groups.

A significant action of cationic biocides is the clustering of anionic lipids, such as phosphatidylglycerol (PG) and CL, which are present in large quantities in bacterial membranes and in the largest percentage in gram-positive cytoplasmic membranes. The clustering of anionic lipids can disrupt existing domains and cause defects at the boundary between the clustered phases. Clustering of lipids can also lead to changes in lipid phase transitions and membrane fluidity and was found from differential scanning calorimetry of cationic oligomers interacting with mixed PE-CL membrane mimics (Epand et al., 2008). Using MD, Alkhalifa et al. observed the clustering of negatively charged lipids (DLMG, DLPG, and TOMCL), in *S. aureus* and *E. coli* simulated membranes, occurring near the ammonium salt of ester- and alkyl-QAC biocides with varying charge (Alkhalifa et al., 2020).

The focus here has been largely on commercially available cationic biocides, in particular some QAC and biguanide derivatives. However, due to the simple structure of the quaternary ammonium group there are an extensive number of possible architectures of compounds containing this moiety which will not be described in detail here. An extensive range of QAC derivatives, including their antimicrobial and antifungal properties, is reviewed in detail by Vereshchagin et al. including a specific mention to those that are commercially used as disinfectants which make up a small portion of studied architectures currently (Vereshchagin et al., 2021).

## Factors Influencing the Activity of Cationic Biocides: Extrinsic Properties

### Concentration

Concentration is a critical factor in determining the outcome of any application of an antimicrobial to a microbial population. Biocides are normally used at concentrations considerably higher than MIC values frequently reported as representing resistance (Gilbert & McBain, 2003). Table 1 illustrates typical in-use concentrations for a variety of medical, community, domestic, and industrial applications for cationic biocides. These values are important when considering reports of biocide resistance in context. In many cases, and as discussed further below, in-use concentrations of biocides can exceed MIC and MBC values (where these are used as the measure) by tens, hundreds or even thousands of times, where the test organisms are described as resistant, tolerant, or of reduced susceptibility. That said, it is important to note that there are instances where MIC/MBC values for clinically relevant strains do approach or exceed recommended use concentrations for some applications (e.g., in food handling environments where safety regulations require maximum biocide residues on equipment and surfaces). Regarding cationic biocides, particular concerns have been raised about chlorhexidine use (Table 1), which has reportedly resulted in acquired resistance to this compound (Kampf, 2016). The review by Kampf summarises relatively high chlorhexidine resistance rates (MICs) in *Enterobacter* spp. (10–75 μg ml^−1^), *Pseudomonas* spp. (2–800 μg ml^−1^), *Proteus* spp. (10–1600 μg ml^−1^), *Providencia* spp. (500-1600 μg ml^−1^), and *Enterococcus* spp. (2–2500 μg ml^−1^), and points out the strong ability of *Acinetobacter* spp., *Klebsiella pneumoniae*, and *Pseudomonas* spp. to adapt to chlorhexidine (Kampf, 2016). In light of this, the author proposes limiting the use of chlorhexidine in applications where it has no demonstrable additional benefit, for example, in soaps for routine hand hygiene, and recommends this approach for biocides in general (Kampf, 2016, 2018). Whilst this approach seems sensible, chlorhexidine remains a highly effective biocide, with typical use concentrations of 2–4% (20 000–40 000 μg ml^−1^, see Table 1). As discussed in more detail below, whilst in some cases there may be an association between biocide use and decreased susceptibility, the occurrence of outcome-altering adaptive decreases in biocide susceptibility are very uncommon.

Many studies of resistance, involve repeated laboratory exposure to sub-inhibitory concentrations of biocides in pure water, thus forcing adaptation of the organisms under conditions that are not representative of real-world exposures. These types of study are often used to indicate the ease and widespread potential for resistance development; however, the clinical relevance of laboratory-adapted strains is debatable. We advocate the increased use of realism-based approaches that reflect more of the complexity of real-world exposure of microbes to biocides, including realistic exposure concentrations and formulations, which should better inform the potential for resistance generation in the environment.

### Formulation

In terms of biocide efficacy and resistance development, another important factor to consider, relevant to commercial biocide preparations, is formulation. Biocides as “actives” are rarely used alone, and instead are formulated in a diverse range of products (Forbes et al., 2017). Co-formulants achieve several objectives including viscosity, odour, and colour and from the perspective of antimicrobial effectiveness include surfactants and sequestrants and may also result in a product that is highly acidic or alkaline and synergistic effects from adding other non-ionic surfactants have been demonstrated (Gomi et al., 2012; Zhang et al., 2017). However, the underlying mechanisms resulting in the increased efficacy due to formulation and the synergistic MOAs remain unclear.

A biocidal product is likely to contain the antimicrobial active compound, or a combination of actives at concentrations that could be over 10 times higher than the MIC or MBC of the target bacteria, along with physical properties such as pH that may be inherently antimicrobial. For example, for preoperative handwashing and MRSA decolonisation, chlorhexidine may be used in aqueous formulations at 4%; for surgical site preparation and the prevention of vascular catheter infections, it can be used at 2% in 70% isopropanol (Horner et al., 2012). The impact of the use of comparatively high concentrations and co-formulants that are intrinsically antimicrobial on the potential to select for resistance, and in the design of laboratory risk assessment method is considered in more detail later in the review.

### pH, Temperature, and Period of Contact

As part of biocidal formulations, pH regulators are often to improve the stability or efficacy of the formulation. However, within the application of a biocide there may often be changes in pH in the environment which differs from the optimum pH of the formulation. This can affect the structure and charge of the active biocidal components. However, many of the pKa values of cationic biocides may be outside the range of standard applications. For example, the calculated pKa of the biguanide group of PHMB is 10.96 (O'Malley et al., 2006) meaning that the groups carry positive charge at physiological pH. At these extreme values it is likely that the effect of pH on the cell will be markedly greater than the effect on the activity of the biocide (O'Malley et al., 2006).

pH is an important factor for the lipids within the biological membranes. For example, the lipid CL has multiple pKa values (pKa_1_ = 2.8, pKa_2_ > 8.5) resulting in changes in structure/conformation with varied environmental pH values. This property of CL is thought to be important for its function. Since CL is present in large quantities in some bacteria membranes, changes in charge density and electrostatic potential of the membrane with pH will have implications to electrostatic interactions with cationic biocides (Sathappa & Alder, 2016).

Temperature can also play a role in the activity of biocides, resulting in changes in solubility and adsorption, thus in biocidal efficacy (Critchley & Bentham, 2009). Temperatures higher than ambient temperature are possible in a large number of applications, for example, the cleaning of linen in a hospital environment (40–65°C), such applications are covered in detail elsewhere (Maillard, 2005). These elevated temperatures may be essential for the antimicrobial effects of the products and will vary between applications. Considerations of the temperature range of biocidal product application on efficacy are therefore important in realism-based approaches.

The exposure time of microorganisms to biocides are also of high importance to their efficacy with inadequate contact times resulting in reduced efficacy. A good example of this is in the application of disinfectant wipes whereby the sporicidal activity of wipes used for 5 minutes was found to be significantly higher than those used for 10 seconds (reflecting an approximated application time). Although it is noted that only 1 of the 10 wipes tested resulted in high sporicidal activity against Clostridium *difficile* (>4 log10) reduction within 5 minutes of contact time (Siani et al., 2011). This also indicates the importance of accurate product labelling with application instructions.

QACs are known for their rapidity of killing, resulting in their widespread use. However, the kill-time is dependent upon several factors including structure, concentration, and temperature. Ioannou et al. performed time-kill studies of *S. aureus* challenged by QAC compounds alkyldimethylbenzylammonium chloride (ADBAC) and DDAC (Ioannou et al., 2007). These revealed the differences in interaction kinetics at different concentrations and temperatures. Profiles of both QACs indicated rapid killing which eventually slowed over time and an increase in the speed of killing from ADBAC with increasing temperature. Uptake isotherms of DDAC indicated a strong initial uptake by bacteria with the possibility of additional binding of biocides to internal targets after membrane lysis. Uptake studies also indicated that at high concentrations, a greater amount of ADBAC remained unbound as compared to DDAC, this could be related to the presence of aggregates of longer-chained BAC present within ADBAC mixtures with lower CMC values. DDAC also inflicted increased and faster leakage of internal cell components compared to ADBAC, indicating greater membrane damage.

The difference in insertion kinetics of alkyl- or ester-QACs was also noted from MD simulations (Alkhalifa et al., 2020) with alkyl ligands penetrating membranes much faster than their ester-counterparts, and a further link suggested between the kinetics of adsorption and MIC (Alkhalifa et al., 2020). The difference in the adsorption profiles of the QACs may be a rational explanation for the difference in time-kill studies and the efficacy of these compounds. The adsorption kinetics of a biocide-surfactant will be affected by its structure as well as the pH, ionic strength, and temperature (Esumi et al., 1996).

## Relating MOA and Structure to Resistance Mechanisms

As discussed in the intrinsic factors section on charge, the efficacy of cationic biocides on bacterial membranes is largely thought to be due to the effect of charge and electrostatic interactions (Alkhalifa et al., 2020). Therefore, the anionic lipid composition of cell membranes will also be important for the efficacy of cationic biocides. This is often given as an explanation for the difference in efficacy of cationic biocides towards gram-negative and gram-positive bacteria due to differences in the ratio of anionic lipids as well as differences in the membrane structures (Russell, 2003). However, this is not the only important feature of cell membranes which can also vary in fatty acid chain length, which alters physical parameters such as fluidity, as well as protein composition. The membrane composition will vary not only between species but also during the lifecycle of bacteria and the processes than underpin these changes are reviewed elsewhere (Zhang & Rock, 2008).

The membrane composition of a cell is highly adaptable and has been shown to vary in response to stress on the cell. These modifications to the cell membrane can include changes in the length and branching of lipids, altering lipid composition (such as net charge) and changes in proteins that modify/protect the membrane (Futoma-Kołoch et al., 2019; Willdigg & Helmann, 2021).

Modifications can be induced by conditions that increase the fluidity of the membrane, such as the presence of biocides (see Fig. 2a). Adjustments to fluidity are important for membrane permeability, protein mobility, and transport processes (Kingston et al., 2011). This adaptation can involve pathways which increase the fatty acid chain lengths, increasing membrane rigidity. However, the mechanisms by which these pathways are triggered by stressors are unclear (Kingston et al., 2011).

Guérin-Méchin et al. reported changes in the fatty acid composition of cells trained with QACs, most noteably modifications in lipid A from lipoplysaccharide (LPS) in *Pseudomonas aeruginosa* K799 (Guérin-Méchin et al., 1999). LPS is highly negatively charged and changes in its structure may have significant impact on interactions with cationic biocides. It was also observed that the fatty acid profile of QAC-trained strains varied depending on the structure of the QACs, again relating possible resistance mechanisms to the intrinsic physical properties or structure of the biocides.

Multidrug transporters in bacteria, located in the cytoplasmic membrane, can remove unwanted/toxic substances. Bacterial resistance to QACs may be manifested in transcriptionally regulated *qac* efflux systems as well as changes to membrane composition discussed above (see Fig. 2). It has also been suggested that the QAC efflux system QacA might be limited to mono- and biscationic QACs (Esumi et al., 1996). Despite the presence of *qac* efflux systems being linked to the decreased bacterial susceptibility of some QACs (Furi et al., 2013), as discussed further below, these studies do not necessarily demonstrate true resistance to the compounds. Due to the membrane disruption MOA of QACs discussed above, increased efflux could be considered to aid in the disruption of membranes by maintaining extracellular concentrations available to adsorb to the membrane, and therefore cannot give a standalone explanation to observed changes in susceptibility to some QAC species, (see Fig. 2b).

 Forman et al. studied 52 novel dye-based QACs (Crystal violet and Malachite green) bearing 1–3 quaternary ammonium groups and 1 or 2 alkyl chain groups with variable length (Forman et al., 2016). Some large differences in MIC were observed between mono- and tri-cationic QACs against bacteria known to bear efflux pumps, with a 500-fold lower MIC (250 μM vs. 0.5 μM) observed for the triscationic QAC. Using these findings, the group arrived at some general trends on the relationships between structure and possible resistance mechanisms. Biocides containing multiple QAC groups (multi-QACs) had no significant difference in antibacterial activity against methicillin-susceptible (MSSA) or community-acquired methicillin-resistant *S. aureus* (CA-MRSA) strains when efflux pump inhibitors were introduced. However, a single-QAC (BAC) showed a fourfold increase in activity against CA-MRSA exposed to efflux pump inhibitors, indicating a significant contribution of efflux pumps on MRSA susceptibility to QACs. These results indicate possible differences in resistance mechanisms due to the charge of QAC-biocides. Both single- and multi-QACs caused similar changes in membrane permeability (assessed by fluorescence spectroscopy) in all strains challenged except for CA-MRSA which had increased permeability for all compounds (Jennings et al., 2017). These results indicate possible biocide selectivity based on the membrane composition and a more complex link between biocidal properties and bacteria susceptibility or resistance mechanisms.

This is an area in which a multi-disciplinary approach, involving biophysical and microbiological studies as well as input from the biocide industry, regulators, and other stakeholders, should prove important in identifying the risks from biocide resistance and mitigating these through an understanding of fundamental structure–function relationships that can inform new product development and improved formulations.

## Microbial Adaptation of Biocides: Defining Biocide Resistance

AMR is arguably best defined as the non-susceptibility of a microorganism to a given treatment (Chen et al., 2013; Dettweiler et al., 2019; Georgiadis et al., 2019; Gilbert & McBain, 2003; Tchapla et al., 2004). Use of the term “resistance” should be guided by conditions and outcomes linked to a real application, and associated with a high likelihood of therapeutic or treatment failure (Gilbert & McBain, 2003). For example, Andersen states “the term antimicrobial resistance should be used to describe situations where: A strain is not killed or inhibited by a concentration attained *in vivo*; A strain is not killed or inhibited by a concentration to which the majority of strains of that organism are susceptible, or Bacterial cells that are not killed or inhibited by a concentration acting upon the majority of cells in that culture,” (Andersen, 2016). For antibiotics, defined breakpoints, including those published by the European Committee on Antimicrobial Susceptibility Testing (EUCAST), are essential in making a meaningful determination of resistance. Breakpoints are linked to the therapeutic outcome such that a bacterium reported as susceptible to a given antibiotic is likely to respond to an antibiotic if appropriately administered (Soares et al., 2020). Such data are particularly important since antibiotics generally have narrow therapeutic windows between an effective dose and toxicity to the host. A relatively small change in antibiotic susceptibility could render a bacterium resistant, meaning that the same appropriate administration of the antibiotic would fail to effectively treat the infection.

Biocides, however, are not generally administered as therapeutics in the same manner as antibiotics. Therefore, breakpoints related to therapeutic outcomes are not meaningfully defined and hence the use of the term “resistance” in the context of biocide applications is problematic. Reports of resistance to biocides, although numerous in the literature, are complicated by the lack of standard terminology, and differences in MOA, application, and concentration between biocides and antibiotics. When reviewing the biocide literature, therefore, particular care must be applied to the term “resistance,” which has been used to describe widely varying observations in biocide susceptibility (Andersen, 2016; Campedelli et al., 2019; Chuanchuen et al., 2005; Russell, 1996). An increase in the MIC or MBC of a biocide to a specific bacterium may not translate to a failure of that product during application, where concentrations can be many times higher, and therefore in such situations, resistance is not the correct term (McBain & Gilbert, 2001).

It is important to examine the susceptibility of bacteria to biocides with realism in mind, incorporating factors which affect biocidal efficacy in the application. These include intrinsic factors such as concentration, stability, formulation, and contact time, as well as extrinsic factors such as the pH, temperature, and the interference of additional organic matter such as proteins. Any of these factors can affect the efficiency of the biocide, possibly exposing the bacteria to nonlethal (or sub-inhibitory) concentrations (Andersen, 2016).

Some bacterial strains exhibit decreased susceptibility to biocides when subjected to concentrations below the MIC for multiple cell cycles (Forbes et al., 2014). (Alkhalifa et al., 2020; Ciumac et al., 2017; McBain et al., 2002). Such laboratory-based adaptation is typically not representative of real-world applications of biocides, or the conditions experienced by microorganisms in the environment. Care should be taken therefore, when drawing conclusions about the potential for resistance during such studies, and we would advocate a more realism-based approach as discussed in more detail below.

It is also important to note that organisms can fail to respond to an antimicrobial for reasons of inherent physiology. Examples include vancomycin insusceptibility in the lactobacilli (Campedelli et al., 2019), the ineffectiveness of the bisphenol biocide triclosan to inactivate certain pseudomonads (Chuanchuen et al., 2005) and the tolerance of mycobacteria to some biocides (Russell, 1996). Such organisms may be referred to as intrinsically resistant.

## Changes in Susceptibility to Cationic Biocides

Where there is a reasonable concern over biocide resistance, application of the precautionary principle is normally the best policy. However, this must be informed by reliable evidence and consider that failure to appropriately deploy biocides where they are indicated can lead to increases in infection rates and in resulting antibiotic use, the main driver of antibiotic resistance.

Questions of resistance risk have been addressed in three main ways: (i) in laboratory studies where bacteria are exposed sub-lethally and repeatedly to a biocide and changes in susceptibility are measured, (ii) where attempts are made to correlate the susceptibility of bacterial isolates to local biocide use, and (iii) where reports of treatment failure are investigated. In addition, mechanistic studies of the possible risks include work that employs genetic manipulation of bacteria, for example, to overexpress efflux pumps implicated in resistance. Reports of potential resistance issues from studies of the first type, involving laboratory exposure, are considerably more numerous than those of environmental surveys, where reports of correlations between biocide use and reduction in susceptibility are noted. Evidence in the third category, relating to overt treatment failures are uncommon (Maillard, 2018). The picture, therefore, remains unclear, resulting in uncertainty about best practice in users and regulators alike. Disinfectants and antiseptics, including QACs and cationic biocides, are widely used in healthcare settings, but acquired resistance in bacteria isolated from clinical samples or the clinical environment has not been commonly observed. A major review of this topic published in 2009 by the European Union's Scientific Committee on Emerging and Newly Identified Health Risks (SCENIHR, 2009) includes the following: “However, unlike antibiotic resistance, the issues relating to biocide resistance are considered to have a very low profile and priority” (Cookson, 2005). Emerging bacterial resistance to biocides has been well described in vitro; but evidence in practice is lacking. Isolates with reduced susceptibility may remain susceptible to clinically used concentrations of the disinfectants (Lear et al., 2006); the concentrations of disinfectants and antiseptic used in practice are substantially higher than the MICs of strains with reduced susceptibility (Cookson, 2005; Lear et al., 2006; Maillard, 2007; Russell, 2002; Weber & Rutala, 2006).

In the subsections that follow, evidence arising from laboratory evolution studies and from studies of clinical and environmental isolates exposed to QACs and cationic biocides are discussed in more detail. The significance of efflux pump activity, often correlated with reduced susceptibility to this class of biocides, is also examined.

### Laboratory-Adapted Strains

Laboratory exposure in risk assessments generally involves growing bacteria in pure culture, in the presence of sublethal concentrations of biocide, most often in simple aqueous solution. The exposure must be sub-effective since non-viable bacteria cannot adapt. Therefore, methods involving repeated exposure to a fraction of the MIC in liquid culture or on agar have been developed (Ledder et al., 2006). Bacteria are highly adaptive and such methods frequently result in changes in susceptibility that may or may not be partially or completely reversible (Knapp et al., 2015). Despite the evidence that bacteria can adapt to certain patterns of biocide exposure *in vitro*, detecting analogous processes in the environment has been considerably more challenging, and the consensus for many years has been that definitive evidence of outcome altering changes in susceptibility is rare (Gilbert & McBain, 2003). Laboratory-based studies, however, indicate that bacteria can adapt to cationic biocides through stepwise exposure to increasing concentrations of the biocide starting from sub-inhibitory levels. Such approaches can produce variants with MICs that are multiples of those of the parent strain. For example, *E. coli* was trained to grow in the presence of 150 μg ml^−1^ BAC, from an MIC of 25 μg ml^−1^, through 24 passages of increasing BAC concentration (Langsrud et al., 2004). The adapted variants had reduced susceptibility to BAC but grew more slowly than control strains, indicating a cost to fitness from the adaptations to BAC. Using a similar approach, *E. coli* K-12 was adapted to MICs for BAC of 80–90 μg ml^−1^ (Bore et al., 2007) and *E. coli* O157 up to MICs of 1000 μg ml^−1^ (Braoudaki & Hilton, 2004). The latter study, and related work, also showed that serovars of *Salmonella enterica* could be trained to BAC adaptation, with an increase in MIC from 4 to 256 μg ml^−1^ for *S. enterica* Virchow (Braoudaki & Hilton, 2004, 2005).

Examples of susceptibility change through *in vitro* studies include work in our own group, where we applied a method based on the deposition of a concentration gradient of a selection of cationic biocides on agar plates using a spiral plater (Moore et al., 2008). Using such methods, bacteria isolated from a domestic sink drain or human skin were exposed to various biocides over 14 passages. In many cases, changes in MIC were relatively small. The highest adapted MBC was approximately 300 μg ml^−1^, lower than many normal biocide use concentrations (Table 1). We revisited this work in 2014, investigating the reversibility of such decreases in biocide susceptibility using similar methods whereby environmental isolates (18 species) were sub-lethally exposed to the cationic microbicides cetrimide, chlorhexidine, PHMB, and the biphenol antimicrobial triclosan, as well as to a cationic antimicrobial peptide (Forbes et al., 2014). Susceptibilities (MICs and MBCs) were determined before and after 10 passages of antimicrobial exposure and after a further 10 passages without antimicrobial. Following exposure, ≥fourfold decreases in susceptibility occurred for cetrimide (5/18 bacteria), antimicrobial peptide apoEdpL-W (7/18), chlorhexidine (8/18), PHMB (8/18), and triclosan (11/18). Of the 34 ≥fourfold increases in the MIC, 15 were fully reversible, 13 were partly reversible, and 6 were non-reversible. Of the 26 ≥fourfold increases in the MBCs, 7 were fully reversible, 14 were partially reversible, and 5 were non-reversible. For cationic biocides tested, the highest MBC observed following adaptation and reversion was 464 μg ml^−1^ (*P. aeruginosa* with cetrimide, unchanged from pre-adaptation value), and typically 232 μg ml^−1^ or lower, again considerably below typical in-use concentrations (see Table 1). The changes in susceptibility for the naturally occluding antimicrobial peptide were generally of similar extent and frequency to the synthetic biocides (Forbes et al., 2014).

Using a broadly similar approach, Condell et al. investigated the tolerance in multidrug-resistant and susceptible *S. enterica* strains to food-grade, commercially available biocide formulations (Condell et al., 2012). They included an assessment of the ability of the bacteria to adapt to these formulations as well as to the active biocidal agents, triclosan, hydrogen peroxide, BAC, and chlorhexidine. What the authors termed cross-tolerance was also investigated. The authors reported that stable tolerance could not be selected for the biocidal formulations although the exposure of *Salmonella* to the biocides in a simple aqueous solution resulted in tolerance for several serotypes of *Salmonella* (Condell et al., 2012). They also reported that “No cross-tolerance to the different biocidal agents or food-grade biocide formulations was observed.” and that “Most tolerant isolates displayed changes in their patterns of susceptibility to antimicrobial compounds” (Condell et al., 2012).

*P. aeruginosa* has been studied in continuous culture in the presence of increasing BAC concentrations (McCay et al., 2010). After 29 generations a stable variant with a similar growth rate to the parent strain was isolated and shown to have an MIC of >350 μg ml^−1^ for BAC (compared to 25 μg ml^−1^ for the parent strain). The authors draw a parallel between chemostat experiments and environmental conditions where microbes might be exposed to a continuum of biocide concentrations (Gilbert & McBain, 2003; McCay et al., 2010). BAC has been detected in environmental waste streams and water at a range of concentrations (Pereira & Tagkopoulos, 2019). However, a chemostat culture of a single bacterium, where BAC is added in a controlled manner, is probably not directly comparable to the complex environmental conditions experienced by a free-living bacterium, which may include competition from other microbes, fluctuating levels of nutrients, oxygen, temperature, hydration, xenobiotics, chemicals, radiation, and a host of other stressors and selection pressures. In a longer-term study, a complex microbial community adapted to BAC in continuous culture over four years demonstrated decreased BAC susceptibility, with MICs of 250 and 460 μg ml^−1^, respectively for dextrin-peptone-BAC and BAC only fed cultures (compared to 100 μg ml^−1^ for the dextrin-peptone fed culture) (Tandukar et al., 2013). Community adaptation to BAC appeared to occur through reductions in microbial diversity and a predominance of intrinsically resistant and BAC-degrading microbes, with *Pseudomonas* spp., prominent amongst them (Tandukar et al., 2013). Gram-positive bacteria have also been subject to laboratory-based adaptation to biocides, notably *S. aureus*. Smith and Hunter worked with a collection of *S. aureus* isolates both sensitive and resistant to antibiotics from hospital and community settings (Smith & Hunter, 2008). The authors reported that three commercial biocides were effective for all isolates, with MBC values 10–1000 times lower than recommended use concentrations. Repeated exposure to increasing levels of biocide resulted in a statistically significant increase in MBC of one of the QAC-based biocides (Trigene, a mixture of BAC and DDAC). The level of increase was unspecified (and presumably did not affect the efficacy of the biocide) but was associated only with isolates that carried *qac* genes encoding efflux pumps (Smith & Hunter, 2008). The association between *qac* genes and biocides has already been touched upon and is discussed further below.

It is evident that exposure of bacteria in pure culture with cationic biocides can result in various types of adaptation. Those that result in adapted bacteria becoming refractory, however, appear to be uncommon. Of more potential concern is co-selection, where decreases in antibiotic susceptibility occur. Due to the relatively narrow therapeutic window, such alterations could affect clinical outcomes if manifested in the environment. The aforementioned studies provide insight into biocide adaptation mechanisms, however, their relevance to how microbes behave towards biocides in the environment is less clear. A key question, therefore, is how well do laboratory simulations reflect conditions in the environment? This will be considered below but first, it is necessary to consider studies of environmental isolates.

### Surveys of Clinical and Environmental Isolates for Biocide Resistance

The BIOHYPO project was a €4.4M European Commission Framework 7 initiative that ran between 2009 and 2012 (European Commission, 2012). The aim was to investigate if biocide use in the food chain would result in a clinically relevant increase in antibiotic resistance in human pathogens. The remit of the project included testing whether biocides select for biocide resistance, if biocides select antibiotic resistance and if this antibiotic resistance would be of clinical relevance. The concerted multi-centre efforts and the resulting publications provide an important resource. The “results in brief” on the EU website state that “Partners found no significant correlation between reduced susceptibility of pathogens to biocides and antibiotic resistance except in the case of chlorhexidine and BAC. However, partners fear that this may change in the future. Type I integrons were found in several human pathogens carrying the genes (*qacA* and *Staphylococcus haemolyticus*(sh)-*fabI* allele) associated with increased biocide and antibiotic resistance” (European Commission, 2020). Data from the project can be found in several papers. Furi et al. screened a large panel of 1602 clinical isolates of *S. aureus* for MIC and MBC values for the biocides BAC and chlorhexidine (Furi et al., 2013). Analysis of the MIC and MBC distributions did not indicate subpopulations with reduced susceptibility to these biocides. In addition, only 14% of efflux mutations selected *in vitro* (by exposure to ethidium bromide and acriflavine, or by serial passage in biocide medium) matched those seen in clinical isolates, leading the authors to question the clinical relevance of such *in vitro* studies (Furi et al., 2013). Furthermore, Morrissey et al., determined the MIC and MBC distributions for four common biocides; triclosan, BAC, chlorhexidine, and sodium hypochlorite for 3319 clinical isolates, which included 1635 *S. aureus*, 901 *Salmonella* spp., 60 *K. pneumoniae*, 54 *Enterobacter* spp., 53 *Enterococcus faecium*, 56 *Enterococcus faecalis*, 368 *E. coli*, and 200 *Candida albicans* (Morrissey et al., 2014). The biocide susceptibility data were used to generate epidemiological cut-off values (ECOFFs) for these strains. ECOFFs represent the upper limit of a normal MIC distribution, such that any isolate presenting an MIC above the ECOFF is considered resistant to the given biocide. This is based on the hypothesis that wild-type microorganisms can be defined by the absence of acquired and mutational mechanisms of resistance to the agent. ECOFFs take no account of biocidal efficacy, and as such a determination of resistance based on an ECOFF does not necessarily predict treatment failure. However, ECOFFs provide a useful measure of changes in biocide susceptibility over time and within microbial populations. For their study with ECOFFs, Morrissey et al. report that in most cases MICs and MBCs followed a normal distribution and that bimodal distributions, that they suggest might indicate the existence of biocide-resistant subpopulations, were observed only for chlorhexidine susceptibility in *Enterobacter* (both MICs and MBCs) and triclosan susceptibility of *Enterobacter* (MBC), *E. coli* (MBC and MIC) and *S. aureus* (MBC and MIC) (Morrissey et al., 2014). Importantly, concerning the cationic biocides BAC and chlorhexidine, the highest MBC reported for any combination of microorganism and agent was 128 μg ml^−1^ (compare with typical use concentrations in Table 1). The authors conclude “that resistance to biocides and, hence any potential association with antibiotic resistance, is uncommon in natural populations of clinically relevant microorganisms” and that “there is no clear evidence that the use of biocides have consistently selected resistant subpopulations presenting MICs above wild-type values, at least by using classical double dilution susceptibility tests.” In another publication associated with the BIOHYPO project, Curiao et al., report the exposure of *E. coli* and *K. pneumoniae* to several biocides in liquid culture, including BAC and chlorhexidine (Curiao et al., 2015). Various changes in susceptibility are reported for differently exposed bacteria with some resulting in decreased fitness. For chlorhexidine and BAC, no MIC of greater than 64 μg ml^−1^ is reported (cf. Table 1).

Not included in the above BIOHYPO studies is the food-borne pathogen *Listeria monocytogenes*. Several studies have highlighted concerns over BAC resistance in *L. monocytogenes* and a possible link to hygiene practices in food-processing plants that might promote the emergence of more virulent strains (Dutta et al., 2013; Kremer et al., 2017; Meier et al., 2017). These studies typically correlate BAC resistance with the presence of plasmid-borne efflux pumps. Such efflux systems are widespread (Dutta et al., 2013; Meier et al., 2017) and can confer low-level resistance to BAC (e.g. growth in the presence of 60 μg ml^−1^ BAC (Kremer et al., 2017)), the driving forces behind their propagation are not clear from correlation studies. Many bacterial isolates from foods and the food-production chain have been reported to exhibit reduced susceptibility to BAC as a commonly used biocide in the food industry. The finding of a high proportion of multiply antibiotic-resistant strains from amongst 378 isolates from 36 organic foods is concerning (Fernández-Fuentes et al., 2012). However, most isolates were actually sensitive to biocides, with 98.2% and 1.8%, respectively having MICs of 10 and 100 μg ml^−1^ for BAC.

A large number of bacteria (1237) isolated from various stages of a goat and lamb meat processing facility were tested for growth in the presence of various biocides and antibiotics. In the case of BAC, resistant strains were defined as those able to grow at the very low concentration of 0.025 μg ml^−1^ (Lavilla Lerma et al., 2013). Principal component analysis in this study implicated BAC and other QACs as the most relevant biocides for resistance (Lavilla Lerma et al., 2013), however, it is difficult to ascertain from the data provided whether the cleaning regime and biocide products used in the plant were effective, or had any effect on the prevention or generation of resistance. In a follow-up study for the same slaughterhouse, ECOFF values for five *Pseudomonas* species were proposed (Lavilla Lerma et al., 2015). In most cases the ECOFF values were not exceeded and in the case of BAC only two isolates each for *Pseudomonas fluorescens* and *Pseudomonas alkylphenolia* were above the ECOFF of 0.25 μg ml^−1^. As only three isolates of *P. fluorescens* and two of *P. alkylphenolia* were included in the analysis it is difficult to say whether these ECOFF values are relevant to the wider population of these species (Lavilla Lerma et al., 2015).

Pseudomonads are persistent and highly adaptive organisms and clinical isolates of *P. aeruginosa* from Japan were reported to have MICs of up to 5000 μg ml^−1^ for BAC as early as 1993 (Kurihara et al., 1993). More recently, a 2018 Iranian study of *P. aeruginosa* has reported isolates from burns with MICs up to 1000 μg ml^−1^ for BAC (Gholamrezazadeh et al., 2018). Romão and co-workers have also studied clinical *P. aeruginosa* isolates in Brazil, utilising a dilution method, involving exposure of culture-contaminated stainless steel carriers to in-use concentrations of a BAC-based commercial product (2000 μg ml^−1^ final concentration) (Romão et al., 2005, 2011). Although not directly comparable with MIC measurements, their work reported reduced susceptibility for 43% of 35 isolates in 2005 (Romão et al., 2005) and 46% of 124 isolates in 2011 (Romão et al., 2011). At present, there is no indication of an upward trend in BAC resistance in populations of *P. aeruginosa* and the propensity for this organism to acquire resistance or pass it on to other species is not clear.

Studies on *Mycobacterium* spp. indicate these organisms have innate recalcitrance towards BAC, although other classes of biocide are highly effective according to Japanese reports (Rikimaru et al., 2000; Shinoda et al., 2016). As with Pseudomonads and other innately recalcitrant organisms, ongoing vigilance around outbreaks and the application of effective disinfection regimens should be implemented.

Comparing isolates from pre- and post-biocide treatment eras may provide useful insights into the potential for the emergence of resistance. A collection of 37 *K. pneumoniae* isolates from 1937 was compared to 39 modern-day isolates and found to have lower-average MIC values for BAC (mode and median 4 μg ml^−1^, compared to mode and median 16 μg ml^−1^) (Wand et al., 2015). As the authors point out, decades-long storage may have altered the traits of the 1937 isolates and therefore affected the biocide susceptibility (Wand et al., 2015). The reasons for the reduction in susceptibility (if any) and potential links to biocide usage were not clear.

Other researchers continue to assess potential links between biocide use and changes in resistance over time. Hijazi et al. for example, present an update to a previous report showing the continuing efficacy of chlorhexidine based infection control measures against *S. aureus* over six years (Hijazi et al., 2016). The aim was to screen *Staphylococcus* isolates collected over another six years in the same Scottish intensive care unit, where chlorhexidine baths form an important component of long-term control of hospital-acquired infections, for the presence of *qacA*/*B* genes implicated in QAC resistance. The authors report “minimal presence of *qacA*/*B* in *S. aureus* strains from screening samples and bacteraemia patients”. However, a high proportion (80%) of *qacA*/*B* carriage is reported for *Staphylococcus epidermidis*, and it is stated that this was associated with reduced susceptibility to chlorhexidine. The authors state that their findings raise concerns over the selection of multidrug resistant strains by chlorhexidine. However, they also state that “chlorhexidine MICs were stable, with no evidence of a steady decrease of susceptibility to chlorhexidine over time, notwithstanding that chlorhexidine had already been in widespread use for 6 years before the start of the present study” (Hijazi et al., 2016).

In an interesting longitudinal survey of bacteria isolated between 1928 and 2012 Hardy et al. report chlorhexidine and octenidine susceptibilities for periods where the use of these biocides, which are commonly used for the decolonization of hospital patients with MRSA, ranged between low use and high use (Hardy et al., 2018). The authors report that the mean MICs and MBCs for chlorhexidine increased over time in bacteria isolated between 1928 and 1953 and bacteria isolated between 2002 and 2012, and then plateaued in organisms isolated between 2013 and 2014 (a period of reduced chlorhexidine use). They also report that susceptibility to octenidine did not change across the first three time-groups (during which time octenidine was not in use) but a significant decrease was observed for bacteria isolated between 2013 and 2014 (corresponding to the introduction of octenidine use). The authors suggest that despite the demonstrable reduction in susceptibility in isolates “the MIC and MBC are still relatively low and significantly below the concentrations at which the antiseptics are used in practice.” They also state that it is unlikely that these isolates will affect clinical efficacy but propose that their emergence indicates that the reduced susceptibility might confer a benefit (Hardy et al., 2018). In general, therefore, based on a sample of the literature where large surveys of bacteria have been laudably conducted, it appears that in some cases there may be an association between biocide use and decreased susceptibility but that outcome-altering adaptive decreases in biocide susceptibility are uncommon in clinical isolates that are considered to be likely to encounter biocidal compounds in the environment. Such observations can be considered in combination with the fact that treatment failures resulting from the proper deployment of biocides attributable to adaptive biocide resistance are not commonly reported.

Other studies involving clinical isolates involving BAC include 894 Asian MRSA isolates (the highest MICs reported were 16 μg ml^−1^) (Noguchi et al., 2005), equal to the proposed ECOFF for *S. aureus* (Morrissey et al., 2014)), vancomycin-sensitive and resistant *E. faecium* from Danish hospitals (a higher proportion of vancomycin-resistant isolates than sensitive isolates had MIC values of 8 μg ml^−1^) (Alotaibi et al., 2017) but none were reported above the proposed ECOFF of 8 μg ml^−1^ for BAC (Morrissey et al., 2014)), and 82 carbapenem-resistant *Acinetobacter baumanii* isolates from intensive care units in China (benzalkonium bromide was tested and MIC values ranged from 32 to 128 μg ml^−1^ for resistant strains, compared to 32 μg ml^−1^ for the sensitive strain; there is currently no proposed ECOFF for this organism).

Correlation analysis is a frequently used tool to establish links between biocide resistance, resistance determinants (such as *qac* genes), and antibiotic susceptibility but causality can generally not be proven. The varying definitions of biocide resistance are also a confounding factor in the context of such correlation-based studies. It appears that environmental studies will continue to play an important role in surveillance but that the risk assessments that are required by regulators and also applied by manufacturers for reasons of diligence ultimately depend on *in vitro* tests, where microorganisms are generally exposed to biocides under controlled conditions.

### Efflux and Resistance to Cationic Biocides

In a review seeking to address whether the wide use of QACs enhance the selection and spread of AMR, Hegstad et al. refer to intrinsic and acquired QAC resistance (Hegstad et al., 2010). Whilst a complete analysis of the papers cited in the Hegstad review is outside the scope of the current article, the first paper cited as demonstrating efflux as a resistance mechanism states that “once a bacterium acquires a gene for a certain multidrug efflux pump or if a silent or weak gene for a multidrug efflux pump is activated, the cell instantly becomes resistant to many antimicrobial agents because multidrug efflux pumps extrude many structurally unrelated antimicrobial agents from cells” (He et al., 2004). However, the highest MICs reported in this paper for BAC and chlorhexidine were 40 μg ml^−1^ and 5 μg ml^−1^, respectively. A paper by Huang et al. is similarly cited. This paper describes the identification and characterisation of a novel chromosomally encoded multidrug resistance efflux protein in *S. aureus*, MdeA (multidrug efflux A) (Huang et al., 2004). It is stated that “when overexpressed, MdeA confers resistance on *S. aureus* to a range of QACs and antibiotics, but not fluoroquinolones” and in the results section, that “both pYH4-mdeA and pYH4-norA conferred resistance to ethidium bromide, benzalkonium chloride, and novobiocin.” The title of the table that is intended to convey this information is “MdeA confers resistance to a range of antimicrobial agents.” In the data presented, the MIC of the mother strain is 1 μg ml^−1^ and for two efflux constructs, MICs of 4 and 8 μg ml^−1^ are reported. Whilst there is an up to eightfold increase in MIC, BAC in real use may be applied at 1500 μg ml^−1^, a concentration 188 times higher than the MIC of the putatively resistant bacterium. Hegstad et al. cite two papers to support the role of reduced permeability or stabilization of the membrane through modifications in lipopolysaccharides in resistance to QAC (Hegstad et al., 2010), namely (Braoudaki & Hilton, 2005); and (Gilbert & Moore, 2005). The highest BAC MIC reported in the former paper is 256 μg ml^−1^, again, considerably lower than the typical in-use concentrations of BAC (Braoudaki & Hilton, 2005) (Table 1). Furthermore, taking the first two papers cited to support the involvement of plasmid-mediated efflux pumps in QAC resistance, Bjorland et al. report that a novel plasmid-borne gene was identified “encoding resistance to quaternary ammonium in three staphylococcal species associated with chronic infections in four horses,” (Bjorland et al., 2003). The highest BAC MIC reported in this paper is 6.0 μg ml^−1^. Ceccarelli et al. did not report QAC susceptibility data (Ceccarelli et al., 2006). Whilst adaptation to QACs may occur, genetic elements, such as efflux genes, characterised as conferring resistance to QACs might be more accurately described as conferring a reduction in QAC susceptibility. As we have already contended above, the upregulation of efflux activity would not appear to offer protection to the microbial outer membrane and thus efflux alone would be unlikely to allow cells to become truly resistant to typical biocide-surfactants. The role of efflux genes, and *qac* genes, in co- or cross-resistance is discussed separately below.

## Co- or Cross-Resistance

Microbial adaptations that promote survival in the presence of biocides also have the potential to confer resistance to other compounds including antibiotics, referred to as cross-resistance. In addition, biocide and antibiotic resistance determinants can co-locate within mobile genetic elements, meaning that the selection of one determinant can co-select for another by the propagation of the entire element, hence co-resistance. Investigations into co- and cross-resistance use similar lines of evidence to those discussed above for resistance to biocides. Many studies have observed reduced susceptibility to antibiotics in bacteria that have been sub-lethally exposed to cationic biocides, including *E. coli* (BAC and chlorhexidine) (Curiao et al., 2015; Guérin et al., 2021; Langsrud et al., 2004), *K. pneumoniae* (BAC and chlorhexidine) (Curiao et al., 2015), *S. enterica* (BAC and QAC-containing commercial products) (Braoudaki & Hilton, 2005; Webber et al., 2015; Whitehead et al., 2011), *S. aureus* (BAC, cetrimide, chlorhexidine) (Huet et al., 2008), *Campylobacter* spp. (BAC, chlorhexidine, cetylpyridinium chloride) (Mavri & Smole Možina, 2013), *P. aeruginosa* (BAC in continuous culture) (McCay et al., 2010) and numerous strains from organic foods (BAC and hexadecylpyridinium chloride) (Gadea et al., 2017). Depending on the parent strain and antibiotics tested the effects on biocide-antibiotic cross-resistance have been variable. In the above cases, the observed antibiotic resistance has been linked to efflux, and in the context of QACs, to the prevalence of efflux pumps encoded by *qac* genes. Efflux pumps often have a broad range of substrates, including both biocides and antibiotics, and as such selection pressure for resistance to one agent may also propagate resistance to another. It should be noted that an active efflux phenotype is likely to have fitness implications for the organism and, as discussed above and further below, the relevance of laboratory-adapted strains to selection pressures in real-world settings is not clear.

A recent study on 205 *L. monocytogenes* isolates from food, animals and the natural environment, reported that exposure to QACs selects for ciprofloxacin resistance (Guérin et al., 2021). For repeated exposure to QACs in a controlled laboratory environment this may be true, however, one cannot conclude from this type of study that biocide use in the food chain is responsible for antibiotic resistance selection. If this were the case, food isolates, which were found to have higher MICs for QACs than animal or environmental isolates, would also be expected to have greater resistance to antibiotics. However, the authors state this was not the case: “Note that the origin of strains did not affect the antibiotic susceptibility profiles amongst the Lm strains panel (data not shown).” Only after laboratory evolution of some strains did a strong correlation with resistance to one antibiotic emerge (ciprofloxacin), whereas for nine others there was no correlation and only a weak correlation with the one other tested (trimetroprim/sulfamethoxazole).

It is reported that long-term exposure to BAC in continuous culture altered the microbial population in a mixed community, originally taken from river sediment (Tandukar et al., 2013). BAC exposure was reported to reduce microbial diversity and enrich a population of BAC degraders, with a higher frequency of efflux pump determinants compared to a non-BAC exposed culture. Increased resistance to penicillin G (MICs >500 μg ml^−1^), tetracycline (MICs up to 250 μg ml^−1^), and ciprofloxacin (MICs up to 18 μg ml^−1^) were evident in BAC-exposed cultures, with efflux determinants such as *sugE, pmpM, mexA, mexE, mexF* likely to be contributing to antibiotic resistance (Konda et al., 2001). As with any study involving highly controlled culture conditions (one-quarter of the culture volume was replaced with fresh medium and BAC, or BAC alone, every three days for over four years), the relevance to real environmental conditions must be considered (Tandukar et al., 2013). Even given that BAC/antibiotic-resistant communities of this type can arise in the environment, questions over their potential to proliferate resistance to biocides and antibiotics remain. Such communities may represent specialist QAC degraders with incidental antibiotic resistance, able to take advantage of conditions that exclude most competitors, and as such they may have limited potential to spread resistance to strains with public health relevance. Further work in this area is needed to ascertain whether or not this is the case.

As an important food-borne pathogen *S. enterica* and its interaction with biocides has been a focus of interest in the field. Whitehead and colleagues, for example, reported that multidrug resistant variants of *S. enterica* serovar Typhimurium could be selected following exposure to biocides (Whitehead et al., 2011). For this study, four commercial biocides with different modes of action were used, including Superkill (BAC, formaldehyde, and glutaraldehyde) and AQAS (an unspecified QAC). After a 5-hour challenge with the recommended in-use concentration of 1% biocide, intact cells were recovered using fluorescence-activated cell sorting (FACS). Stably multidrug resistant variants were recovered for the BAC-containing product, although cells recovered after AQAS treatment were not culturable. Low-level exposure to the biocides (0.005%) did not alter the antibiotic susceptibility or pre-dispose the cells to biocide survival in a repeat challenge. The authors discuss not having been able to isolate biocide-resistant *Salmonella* variants in previous work using conventional culture-based techniques and propose FACS as a sensitive method for isolation of biocide-resistant bacteria. However, none of the multidrug resistant variants isolated had any change in MIC to the biocides they were challenged with (Whitehead et al., 2011). Individual microbial cells in any given population might be expected to survive with low frequency following a biocide challenge. Whilst FACS might provide a sensitive tool for isolating these survivors, it is questionable whether the pre-culturing, biocide challenge, screening, and recovery conditions are relevant to microbial population dynamics and the development of resistance in real-world clinical or food-processing environments.

In terms of environmental isolates, the findings of the BIOHYPO project again provide useful data in terms of cross-resistance. Morrissey et al. reported that biocide-resistant mutants were rare and that “this would imply that co-selection or cross-selection of antibiotic resistance should also be a rare event in natural populations.” Oggioni et al. reported on an MIC-based survey of over 1600 clinical *S. aureus* testing potential correlation between antibiotic sensitivity and susceptibility profiles of BAC, chlorhexidine, triclosan, and sodium hypochlorite (Oggioni et al., 2015). Whilst the authors report correlation coefficients for MICs of BAC and chlorhexidine above 0.4, for susceptibility to quinolones, beta-lactams, and macrolides, they conclude that their data “do not support any selective pressure for association between biocides and antibiotics resistance and furthermore do not allow for a defined risk evaluation for some of the compounds” and that “These data hence infer that biocide selection for antibiotic resistance has had so far a less significant impact than feared” (Oggioni et al., 2015).

The significance of co-selection in the proliferation of antibiotic and biocide resistance, and the role of cationic biocides therein, is debatable. Several studies have identified frequent occurrences of *qac* genes in Class I integrons, concluding from this that QACs can co-select for antibiotic resistance because Class I integrons are known to accumulate and transmit gene cassettes for antibiotic resistance (Gaze et al., 2005; Gillings et al., 2009). The co-occurrence of biocide and antibiotic resistance determinants on mobilisable plasmids also gives the potential for co-selection. A 2015 study of publicly available sequence databases identified significant co-occurrences of biocide/metal resistance genes and antibiotic resistance genes on bacterial plasmids and genomes (Pal et al., 2015). The only biocide resistance gene commonly occurring with antibiotic resistance genes was *qacEΔ1*, which given its origins and close association with *sulI* is not surprising (Paulsen et al., 1993). Sulphonamide resistance seems likely to be the main selection or co-selection determinant in this case. If *qac* genes were significant determinants for co-selection of antibiotic resistance genes, a higher frequency of co-occurrence might be expected for those *qac* genes, such as *qacE*, that impart a higher level of resistance, but this does not seem to be the case. One caveat to this is that other resistance mechanisms, including efflux pumps not annotated as *qac* genes, may act on biocides and thus exert sufficient pressure for co-selection, although there is no clear evidence for this at present.

## Towards Greater Realism in Risk Assessment

The potential risks of biocide use must be evaluated seriously using a range of techniques to best reproduce likely exposure scenarios. Whilst reductions in biocide susceptibility have been frequently reported following laboratory exposure, it is arguable that few studies have reproduced conditions that reflect the real-world deployment of the active compounds. Whilst pure culture laboratory exposure can be useful to ascertain what could potentially happen in theoretical scenarios, it is unrealistic because (i) in the real world, bacteria rarely grow in pure culture; (ii) antimicrobials are almost always deployed in formulations containing a variety of excipients (some of which potentiate the activity of the actives); and (iii) bacteria in the real world are subject to a range of physical and chemical stresses, including nutrient limitation. Conditions analogous to batch culture growth in complex laboratory media are uncommon. Many of the publications that have demonstrated susceptibility changes to biocides using highly selective conditions that do not reflect normal use at the primary site of application have reported that adaptation is unstable, suggestive of phenotypic adaptation. For example, Jones et al. used a batch culture system to expose *P. aeruginosa* to a QAC and observed that decreases in susceptibly once acquired, were gradually lost (Jones, Herd, & Christie, 1989). Méchin et al. similarly noted that susceptibility decreases to a QAC were reversed when the cells were transferred to biocide-free medium (Méchin et al., 1999).

The main factors that separate many reports used in risk assessment from real-world biocide use are as follows: (i) Concentration. Necessarily sub-lethal *in vitro* for resistance to be selected, whereas applications must be effectively bactericidal/bacteriostatic. (ii) Bioburden. Almost always high in *in vitro* studies. Adaptation may be a rare event at the microbial population level, and thus, high bioburdens increase the potential for susceptibility changes, to give the investigators a positive observation to publish. Bioburdens are generally comparatively very low in preservation. (iii) Formulation. Normally simple aqueous solutions in *in vitro* studies whereas biocides are often formulated in real use. Where applied, this can significantly increase antimicrobial potency and mitigates the risk of resistance. (iv) Competitive fitness of adapted organisms (i.e., the metabolic burden of adaptation) where susceptibility changes have been generated *in vitro*. If risk-assessment studies applied biocides at in-use concentrations (e.g., 1500–2000 μg ml^−1^, cf. Table 1) resistance would be an unlikely outcome.

Forbes and colleagues carried out a series of “realism-based” studies, intended to improve the accuracy of risk assessments by considering how bacteria are exposed to biocides in the domestic environment (Forbes et al., 2017). They hypothesised that the formulation of biocides could be an important variable. The effect of formulation on antimicrobial activity and the induction of bacterial insusceptibility in several bacteria including *P. aeruginosa*, was assessed after exposure over 14 passages to 8 biocides including BAC, in formulation with various excipients. Susceptibilities were also assessed following 14 passages in biocide-free medium (Cowley et al., 2015). The following observations were made: (i) The biocides were over 10-fold more potent in the formulation (i.e., as they are normally deployed in real life) than in simple aqueous solution; ii) “After exposure to the antimicrobial compounds, of 72 combinations of microbicide and bacterium there were 19 ≥4-fold (mean, 8-fold) increases in MIC for non-formulated and 8 ≥4-fold (mean, 2-fold) increases in MIC for formulated microbicides”. In most cases, a twofold change in susceptibility will not result in outcome altering resistance; (iii) Susceptibility decreases fully or partially reverted to pre-exposure values for 49% of MICs and 72% of MBCs after further passage. Formulation substantially mitigated the susceptibility effect of BAC exposure on the susceptibility of *P. aeruginosa*; and (iv) It was concluded that formulated microbicides exhibit significantly greater antibacterial potency than unformulated actives, and that susceptibility decreases after repeated exposure was lower in frequency and extent for formulated biocides. This study illustrates the principle that testing pure culture using biocides such as QACs in simple aqueous solution does not accurately quantify the risk of biocide use and could overestimate the potential for and the extent of likely risks. A related paper Forbes et al. assessed bacterial antibiotic susceptibility in biocide-exposed bacteria. Statistically significant decreases in antibiotic susceptibility occurred for 12% of bacteria after exposure to biocides in formulation and 20% of bacteria after exposure to microbicides in aqueous solutions (Forbes et al., 2016). Importantly, of the combinations of a bacterium and an antibiotic for which British Society for Antimicrobial Chemotherapy breakpoints are available, none became resistant.

To observe the development of biocide resistance in the laboratory, and therefore draw inferences about it, studies invariably use non-formulated active compounds at sub-inhibitory concentrations, precisely because the use of real-world biocides at in-use concentrations are unlikely to generate resistance. As an example to illustrate this point, a 2010 study by McCay et al. grew *P. aeruginosa* in continuous culture in the presence of BAC at 12.5 μg ml^−1^ (50% of the MIC of the test organism) (McCay et al., 2010). Assuming a typical in-use concentration of 1500 μg ml^−1^ (cf. Table 1), the exposure concentration would be >120 times lower than that for the primary application of a disinfectant. Also, this does not factor in other mitigating effects, which include formulation, homogeneity, low-density microbial challenge, and growth substrate environment, any of which could substantially affect the potential for resistance development. Furthermore, if simulating disinfection in the laboratory, it would be reasonable to add a realistic (i.e., comparatively low) microbial challenge to the product, which would further mitigate the risk of resistance. In the McCay et al. study, 480 ml of medium was inoculated with 20 ml of an overnight culture and allowed to grow for six generations before the addition of BAC (McCay et al., 2010). Although the precise density of the culture at BAC addition is not known, the bioburden is likely to be very high, and not representative of a real-world disinfection scenario. Had the initial inoculum been challenged with, say, 1500 μg ml^−1^ BAC it would likely have been inactivated and resistance development not observed. If, in this scenario, a bacterium did acquire significantly reduced QAC susceptibility, data in the McCay et al. study (the highest BAC MIC reported was around 350 μg ml^−1^), suggest that it would not be able to proliferate, since in most disinfection scenarios the organism remained susceptible to in-use concentrations (Table 1). McCay et al. also observed that, under glucose limitation, a BAC-adapted variant lost its selective advantage in the presence of BAC, suggesting that it may also be uncompetitive in the general environment were it to be released (McCay et al., 2010).

## Conclusions

There are many situations in which there is a clear need to control or inactivate microorganisms. These include hygiene in healthcare, farming, food processing, domestic and industrial applications, and in the preservation of products. Appropriate control of microbial growth is a key facet of hygiene and a route through which the incidence of infection can be minimised, thus decreasing the burden on antibiotic use. Biocidal compounds represent an important tool through which this can be achieved but their appropriate deployment depends on the understanding of the MOA as well as the risks of resistance.

Many reports of resistance to cationic biocides have focused on the involvement of efflux pumps and in particular those encoded by *qac* genes. Efflux mechanisms may offer only partial protection to the primary target of cationic biocides, the outer membrane, and as such may be limited to providing low-level resistance to biocides. Therefore, a fuller understanding of potential resistance risks must include a deeper understanding of the fundamentals of biocide–membrane interactions, for example through the study of model membrane systems. The physicochemical properties of biocides (such as the charge and hydrophobicity) affect their efficacy and modes of action. In particular, the chemical properties of surfactant-biocides can drastically change their physical properties, such as CMC, influencing how they interact with components of biological membranes, and in turn their MICs. Biophysical studies of model membrane systems, allied with microbiological studies of biocidal products, provide an avenue to assess the risks of membrane-targeted biocide resistance and allow the possibility to reformulate products towards resistance risk mitigation.

To date, several lines of evidence have been used to determine the risks of biocide resistance and co-resistance including laboratory exposure studies, correlation analysis, and reports of treatment failure. Establishing a direct link between biocide use and resistance has been challenging and there has often been a lack of agreement between *in vitro* data and observation from environments where biocidal compounds are deployed. Microorganisms have evolved to adapt to survive inimical conditions so it is unsurprising that in some cases organisms can exhibit reduced susceptibility following sub-lethal exposure to biocidal compounds. There is a lack of clarity and consistency of the definition of resistance, with organisms described as biocide-resistant retaining susceptibility to real applications. The precautionary principle behind the need to control the deployment of biocidal compounds should be balanced against the demonstrable benefits of microbial control, which offer the opportunity to minimise the deployment of antibiotics, which are generally accepted to be the main driver of AMR. As with any class of antimicrobial compound, however, biocides should be deployed where there is a well-evidenced benefit.

We have argued above for increased realism in the study of potential resistance to biocides, including realistic use concentrations of biocides, formulations, and bioburdens of test organism, as well as conditions more representative of the real-world environment. Whilst acknowledging that laboratory-based risk assessment studies can never fully reproduce the complexities of real-world exposure conditions, we maintain that more work is needed to develop greater realism and a generally accepted framework for resistance risk assessment. Against the background of increasing regulatory pressure on biocide producers, to provide evidence of resistance development potential from their products, it is an important moment for stakeholders and regulators to work together to improve understanding and to develop biocide formulations, standards and practices that limit resistance.
